# Genetic variation in antidiabetic drug targets: associations with Parkinson’s disease risk and age at onset

**DOI:** 10.1038/s41531-026-01398-5

**Published:** 2026-05-28

**Authors:** Katalin Vincze, Agnieszka Szwajda, Alexander Ploner, Robert Karlsson, Xiaoying Kang, Bowen Tang, Chenxi Qin, Cloé Domenighetti, Pierre-Emmanuel Sugier, Ashwin Ashok Kumar Sreelatha, Claudia Schulte, Berta Portugal, Patrick May, Dheeraj Reddy Bobbili, Milena Radivojkov-Blagojevic, Peter Lichtner, Andrew B. Singleton, Dena G. Hernandez, Connor Edsall, George D. Mellick, Alexander Zimprich, Walter Pirker, Ekaterina Rogaeva, Anthony E. Lang, Sulev Koks, Pille Taba, Suzanne Lesage, Alexis Brice, Jean-Christophe Corvol, Marie-Christine Chartier-Harlin, Eugénie Mutez, Kathrin Brockmann, Angela B. Deutschlander, Lena F. Burbulla, Georges M. Hadjigeorgiou, Efthimos Dardiotis, Leonidas Stefanis, Athina Maria Simitsi, Enza Maria Valente, Simona Petrucci, Letizia Straniero, Anna Zecchinelli, Gianni Pezzoli, Laura Brighina, Carlo Ferrarese, Grazia Annesi, Andrea Quattrone, Monica Gagliardi, Hirotaka Matsuo, Akiyoshi Nakayama, Nobutaka Hattori, Kenya Nishioka, Sun Ju Chung, Yun Joong Kim, Pierre Kolber, Bart PC Van de Warrenburg, Bastiaan R. Bloem, Mathias Toft, Lasse Pihlstrøm, Leonor Correia Guedes, Joaquim J. Ferreira, Soraya Bardien, Jonathan Carr, Eduardo Tolosa, Mario Ezquerra, Pau Pastor, Caroline Ran, Andrea C. Belin, Andreas Puschmann, Carl E. Clarke, Karen E. Morrison, Manuela Tan, Dimitri Krainc, Matt J. Farrer, Zied Landoulsi, Rejko Kruger, Thomas Gasser, Alexis Elbaz, Manu Sharma, Nancy L. Pedersen, Sofia Carlsson, Sara Hägg, Karin Wirdefeldt, Cloé Domenighetti, Pierre-Emmanuel Sugier, Ashwin Ashok Kumar Sreelatha, Claudia Schulte, Berta Portugal, Patrick May, Dheeraj Reddy Bobbili, Milena Radivojkov-Blagojevic, Peter Lichtner, Andrew B. Singleton, Dena G. Hernandez, Connor Edsall, George D. Mellick, Alexander Zimprich, Walter Pirker, Ekaterina Rogaeva, Anthony E. Lang, Sulev Koks, Pille Taba, Suzanne Lesage, Alexis Brice, Jean-Christophe Corvol, Marie-Christine Chartier-Harlin, Eugénie Mutez, Kathrin Brockmann, Angela B. Deutschlander, Lena F. Burbulla, Georges M. Hadjigeorgiou, Efthimos Dardiotis, Leonidas Stefanis, Athina Maria Simitsi, Enza Maria Valente, Simona Petrucci, Letizia Straniero, Anna Zecchinelli, Gianni Pezzoli, Laura Brighina, Carlo Ferrarese, Grazia Annesi, Andrea Quattrone, Monica Gagliardi, Hirotaka Matsuo, Akiyoshi Nakayama, Nobutaka Hattori, Kenya Nishioka, Sun Ju Chung, Yun Joong Kim, Pierre Kolber, Bart PC Van de Warrenburg, Bastiaan R. Bloem, Mathias Toft, Lasse Pihlstrøm, Leonor Correia Guedes, Joaquim J. Ferreira, Soraya Bardien, Jonathan Carr, Eduardo Tolosa, Mario Ezquerra, Pau Pastor, Caroline Ran, Andrea C. Belin, Andreas Puschmann, Carl E. Clarke, Karen E. Morrison, Manuela Tan, Dimitri Krainc, Matt J. Farrer, Zied Landoulsi, Rejko Kruger, Thomas Gasser, Alexis Elbaz, Manu Sharma, Karin Wirdefeldt

**Affiliations:** 1 Department of Medical Epidemiology and Biostatistics, Karolinska Institutet, Stockholm, Sweden; 2 Karolinska Institutet, Institute of Environmental Medicine, Stockholm, Sweden; 3 Gustave Roussy, University of Paris-Saclay, UVSQ, Inserm, Villejuif, France; 4 University of Tübingen, Institute for Clinical Epidemiology and Applied Biometry, Centre for Genetic Epidemiology, Tübingen, Germany; 5 Department for Neurodegenerative Diseases, University of Tübingen, Hertie Institute for Clinical Brain Research, Tübingen, Germany; 6 German Center for Neurodegenerative Diseases, Tübingen, Germany; 7 University of Luxembourg, Luxembourg Centre for Systems Biomedicine (LCSB), Translational Neuroscience, Luxembourg, Luxembourg; 8 Luxembourg Centre for Systems Biomedicine (LCSB), University of Luxembourg, Luxembourg, Luxembourg; 9 Helmholtz Zentrum München, Institute of Human Genetics, Munich, Germany; 10 National Institutes of Health, Laboratory of Neurogenetics, Molecular Genetics Section, Bethesda, MD, USA; 11 National Institutes of Health, Center For Alzheimer’s and Related Dementias, Bethesda, MD, USA; 12 Griffith Institute for Drug Discovery, Griffith University, Brisbane, QLD, Australia; 13 Department of Neurology, Medical University of Vienna, Vienna, Austria; 14 Department of Neurology, Klinik Ottakring, Vienna, Austria; 15 Tanz Centre for Research in Neurodegenerative Diseases, University of Toronto, Toronto, ON, Canada; 16 Toronto Western Hospital, Edmond J. Safra Program in Parkinson’s Disease, Morton and Gloria Shulman Movement Disorders Clinic, Toronto, ON, Canada; 17 Division of Neurology, University of Toronto, Toronto, ON, Canada; 18 Centre for Molecular Medicine and Innovative Therapeutics, Murdoch University, Perth, WA, Australia; 19 Perron Institute for Neurological and Translational Science, Nedlands, WA, Australia; 20 Institute of Clinical Medicine, University of Tartu, Faculty of Medicine, Tartu, Estonia; 21 Clinic of Neurology, Tartu University Hospital, Tartu, Estonia; 22 Department of Neurologie, Sorbonne University, Institut du Cerveau - Paris Brain Institute - ICM, INSERM, CNRS, Assistance Publique Hôpitaux de Paris, Paris, France; 23 Assistance Publique Hôpitaux de Paris, Department of Neurology, CIC Neurosciences, Paris, France; 24 Centre de Recherche Lille Neurosciences & Cognition, University of Lille, Inserm, Lille, France; 25 Department of Neurology, Ludwig-Maximilians-Universität München, Munich, Germany; 26 Department of Neurology, Max Planck Institute of Psychiatry, Munich, Germany; 27 Metabolic Biochemistry, Ludwig-Maximilians-Universität München, Faculty of Medicine, Biomedical Center, Munich, Germany; 28 Department of Neurology, University of Cyprus, Medical School, Nicosia, Cyprus; 29 Laboratory of Neurogenetics, University Hospital of Larissa, University of Thessaly, Department of Neurology, Larissa, Greece; 30 Biomedical Research Foundation of the Academy of Athens, Experimental Surgery and Translational Research, Center of Clinical Research, Athens, Greece; 31 1st Department of Neurology, National and Kapodistrian University of Athens, Medical School, Eginition Hospital, Athens, Greece; 32 Department of Molecular Medicine, University of Pavia, Pavia, Italy; 33 Mondino Foundation, Istituto di Ricovero e Cura a Carattere Scientifico (IRCCS), Pavia, Italy; 34 Department of Clinical and Molecular Medicine, Sapienza University of Rome, Rome, Italy; 35 UOC Medical Genetics and Advanced Cell Diagnostics, S. Andrea University Hospital, Rome, Italy; 36 Department of Biomedical Sciences, Humanitas University, Milan, Italy; 37 Azienda Socio Sanitaria Territoriale (ASST) Gaetano Pini/CTO, Parkinson Institute, Milan, Italy; 38 Fontazione Grigioni, Parkinson Institute, Milan, Italy; 39 Department of Neurology, Fondazione IRCCS San Gerardo dei Tintori, Monza, Italy; 40 Department of Medicine and Surgery and Milan Center for Neuroscience, University of Milano-Bicocca, Milan, Italy; 41 National Research Council, Cosenza, Italy; 42 Department of Medical and Surgical Sciences, Institute of Neurology, Magna Graecia University, Catanzaro, Italy; 43 Department of Medical and Surgical Sciences, Neuroscience Research Center, Magna Graecia University, Catanzaro, Italy; 44 Department of Integrative Physiology and Bio-Nano Medicine, National Defense Medical College, Saitama, Japan; 45 Department of Neurology, Juntendo University School of Medicine, Bunkyo-ku, Tokyo, Japan; 46 Department of Neurology, University of Ulsan College of Medicine, Asan Medical Center, Seoul, South Korea; 47 Department of Neurology, Yonsei University College of Medicine, Seoul, South Korea; 48 Centre Hospitalier de Luxembourg, Neurology, Luxembourg, Luxembourg; 49 Department of Neurology, Radboud University Nijmegen Medical Centre, Donders Institute for Brain, Cognition and Behaviour, Nijmegen, Netherlands; 50 Department of Neurology, Oslo University Hospital, Oslo, Norway; 51 Instituto de Medicina Molecular João Lobo Antunes, University of Lisbon, Faculty of Medicine, Lisbon, Portugal; 52 Department of Neurosciences and Mental Health, Neurology, Centro Hospitalar Lisboa Norte, Hospital de Santa Maria, Lisbon, Portugal; 53 Laboratory of Clinical Pharmacology and Therapeutics, University of Lisbon, Faculty of Medicine, Lisbon, Portugal; 54 Department of Biomedical Sciences, Division of Molecular Biology and Human Genetics,Stellenbosch University, Faculty of Medicine and Health Sciences, Cape Town, South Africa; 55 South African Medical Research Council/Stellenbosch University Genomics of Brain Disorders Research Unit, Stellenbosch University, Cape Town, South Africa; 56 Department of Medicine, Division of Neurology, Stellenbosch University, Faculty of Medicine and Health Sciences, Stellenbosch, South Africa; 57 Parkinson’s disease & Movement Disorders Unit, University of Barcelona, Institut d’Investigacions Biomèdiques August Pi i Sunyer (IDIBAPS), Hospital Clínic de Barcelona, Neurology Service, Barcelona, Spain; 58 Centro de Investigación Biomédica en Red, Enfermedades Neurodegenerativas, Barcelona, Spain; 59 Lab of Parkinson Disease and Other Neurodegenerative Movement Disorders, University of Barcelona, Institut de Neurociències, Institut d’Investigacions Biomèdiques August Pi i Sunyer (IDIBAPS), Barcelona, Spain; 60 Fundació per la Recerca Biomèdica i Social Mútua Terrassa, Barcelona, Spain; 61 Department of Neurology, Movement Disorders Unit, University Hospital Mútua de Terrassa, Terrassa, Spain; 62 Department of Neuroscience, Karolinska Institutet, Stockholm, Sweden; 63 Department of Clinical Sciences Lund, Division of Neurology, Lund University, Lund, Sweden; 64 Department of Neurology, Skåne University Hospital, Lund, Sweden; 65 University of Birmingham, Birmingham, United Kingdom; 66 Sandwell & West Birmingham Hospitals NHS Trust, West Bromwich, United Kingdom; 67 Health and Life Sciences, Faculty of Medicine, Queens University, Belfast, United Kingdom; 68 Department of Clinical and Movement Neurosciences, University College London, UCL Queen Square Institute of Neurology, London, United Kingdom; 69 Department of Neurology, Northwestern University Feinberg School of Medicine, Chicago, IL, USA; 70 Department of Neurology, McKnight Brain Institute, University of Florida, Gainesville, FL, USA; 71 Luxembourg Institute of Health, Transversal Translational Medicine, Strassen, Luxembourg; 72 Department of Clinical Neuroscience, Karolinska Institutet, Stockholm, Sweden

**Keywords:** Diseases, Genetics, Medical research, Neurology

## Abstract

To investigate whether antidiabetic drugs have a biological basis to be repurposed in PD prevention, we applied a drug target Mendelian randomization framework to assess associations between genetic variation in antidiabetic drug targets and PD risk or age at onset (AAO). Instrumental variables (IVs) were derived from GWAS summary statistics on fasting glucose (FG), glycated hemoglobin (HbA1c), and gene expression data from GTEx. Apart from SGLT2 inhibitors, all other antidiabetic drugs of interest could be instrumented through our methods. Positive and negative control analyses were carried out to validate 20 IVs in the FG arm and 23 IVs in the HbA1c arm. DPP-4 inhibitors failed the positive control. GWAS summary statistics for PD risk and AAO data were sourced from the IPDGC and COURAGE-PD consortia, resulting in 42 083 cases/457 090 controls for risk and 37 103 PD cases for AAO. MR analyses showed no significant associations across consortia or in meta-analysis. These findings do not support a causal role of genetic variation in antidiabetic drug targets in PD risk or AAO.

## Introduction

Parkinson’s disease (PD) is a neurodegenerative disorder affecting around 12 million people globally^1^ with no disease-modifying treatments available^2^. PD has a moderate genetic component, with heritability estimated at 16–36% (excluding monogenic mutations, which have higher penetrance, and account for <2–5% of cases)^2,3^. The pathophysiology of PD involves alpha-synuclein aggregation, neuroinflammation, vesicle and synaptic transport impairment and dysfunction of mitochondria^2^. Some of these mechanisms are not unique to PD, and using drugs already proven to work for other diseases is a promising option in PD treatment.

Type 2 diabetes (T2D) and PD share common molecular^4^ and biological pathways, such as protein accumulation, lysosomal and mitochondrial dysfunction, and chronic systemic inflammation^5–8^. Drug repurposing or repositioning uses already existing and safety-tested drugs for new indications^9^. This is preferable compared to developing new substances due to shorter timeline for development and approval^9^. Considering that T2D and PD have biological processes in common, repurposing antidiabetic medications for PD treatment or prevention has been suggested^8^. Both traditional and recently developed antidiabetic drugs have lately been a focus of repurposing efforts for neurodegenerative diseases^10–12^, including PD^8,12^.

Metformin has been associated with a lower risk of PD and other neurodegenerative diseases by a range of observational studies^13–15^. GLP-1 receptor agonists and DPP-4 inhibitors have also shown promising PD prevention results in a cohort study of diabetic patients^16^. According to a 2024 update of the results from PD drug clinical trials, there are 5 ongoing trials of GLP-1 receptor agonists as disease-modifying treatment for PD^17^. On the other hand, a case-control study of PD risk in diabetes patients found no association between a range of diabetes medications (insulins, sulfonylureas, DPP-4 inhibitors, GLP-1 receptor agonists and metformin) and PD, apart from thiazolidinediones – which was associated with a reduced PD risk^18^. Additionally, SGLT2 inhibitors have been discussed as a potentially viable repurposing alternative^19^. Thus, observational evidence on potential repurposing of antidiabetic drugs in PD prevention is conflicting.

Mendelian randomization (MR) is an analytical method that uses genetic variants (such as single-nucleotide polymorphisms, SNPs) as instrumental variables (IVs) to proxy environmental exposures, minimizing confounding and reverse causation in observational studies^20,21^. Drug target MR is a specialized form of MR often applied with the aim to explore repurposing opportunities^20^. For IVs to be valid in drug target MR, they should meet three key assumptions: 1) relevance (the IVs are associated with the exposure), 2) independence (IVs are not associated with confounders), and 3) exclusion restriction (no horizontal pleiotropy, meaning IVs influence the outcome only through the drug target)^20^.

MR evidence on potential repurposing of antidiabetic drugs in PD prevention is inconclusive. Metformin has been linked to reduced PD risk in a drug target MR study^22^, hypothetically due to mechanisms such as neuroprotection^22^ or regulation of mitochondrial activity^23^. However, both metformin, GLP-1 receptor agonists, and insulin were linked to worsened motor symptoms in another drug target MR study^24^. SGLT2 inhibitors have also been associated with increased risk for PD in a drug target MR study^25^. Another MR study suggested sulfonylureas as an alternative for PD prevention^26^, while a recent study using the same outcome data source suggested no repurposing benefit of antidiabetic drugs in general for PD prevention or treatment^27^. Limitations of previous drug target MR studies of antidiabetic drugs in PD include that they often relied solely on existing literature or the same resources to identify and validate IVs, they focused on only a subset of antidiabetic drugs and predominantly used the same genome-wide association studies (GWAS) for PD-related outcomes.

The aim of the present study was to investigate whether antidiabetic drugs have a biological basis for repurposing as primary preventive agents against PD, applying a comprehensive IV selection process and leveraging a large-scale meta-analysis of genetic data on PD risk and AAO that had not been previously utilized for the topic of interest from the Comprehensive Unbiased Risk Factor Assessment for Genetics and Environment in Parkinson’s Disease (COURAGE-PD) consortium^28–30^ in addition to the International Parkinson’s Disease Genomics Consortium (IPDGC) data^31,32^. This resulted in a sample of 42 083 cases and 457 090 controls for PD risk, and 37 103 cases for PD AAO.

## Results

### Selection of IVs

The number of IVs (SNPs) per drug class identified varied between zero (no IVs identified) and 22 (Table 1). Our biologically informed selection criteria were based on statistical significance from the GWAS summary statistics on fasting glucose (FG) or glycated hemoglobin (HbA1c) (comprising two different arms of the analysis) and gene expression data (expression quantitative trait loci – eQTL). Metformin was instrumented by one IV in the FG arm, insulin/insulin analogues by two IVs in the FG arm and one in the HbA1c arm, GLP-1 receptor agonists by three IVs in each arm, and sulfonylureas by four in the FG arm and two in the HbA1c arm. Thiazolidinediones were instrumented with the most IVs both in the FG arm (*N* = 17) and in the HbA1c arm of the study (*N* = 22). For SGLT2 inhibitors in both arms and metformin in the HbA1c arm, no IVs were identified due to SGLT2 inhibitor IV candidates not fulfilling the filtering criteria in the downstream GWASes. IVs and their corresponding genes with flanking regions per drug class are detailed in Table 1. The number of IVs was the same as the number of genes in all cases. IV alleles were harmonized such that effect alleles corresponded to negative effect estimates. F statistics were calculated for all IV sets, and results (F > 10) indicated that the IV sets selected had appropriate instrument strength (Supplementary Tables 1, 2).

**Table 1:** Selected instrumental variables per drug class depending on the downstream biomarker (fasting glucose and glycated hemoglobin)

Drug	Fasting glucose arm			HbA1c arm		
	Gene	Gene location (in GRCh37 from Ensembl) without the +/− 2.5k base pairs flanking region	Variants	Gene	Gene location (in GRCh37 from Ensembl) without the +/− 2.5k base pairs flanking region	Variants
SGLT2 inhibitors	0	0	0	0	0	0
Metformin	IGF1R	chr 15:99192200-99507759	rs6598541	0	0	0
DPP-4 inhibitors	GLP1R	chr 6:39016574-39055519	rs6923761	DPP8	chr 15:65734801-65810042	rs11636148
Insulin/insulin analogues	IGF1R, INS	chr 15: 99192200-99507759, chr 11:2181009-2182571	rs6598541, rs3842754	INS	chr 11:2181009-2182571	rs3842756
GLP-1 receptor agonists	DGAT1, GLP1R, INS	chr 8:145539954-145550573, chr 6:39016574-39055519, chr 11:2181009-2182571	rs3757974, rs6923761, rs3842754	CDH1, INS, SREBF1	chr 16:68771128-68869451, chr 11:2181009-2182571, chr 17:17713713-17740325	rs7198799, rs3842756, rs9894257
Sulfonylureas	ABCB11, CYP1A2, INS, TPD52	chr 2:169779448-169887832, chr 15:75041185-75048543, chr 11:2181009-2182571, chr 8:80870571-81143467	rs557462, rs2960192, rs3842754, rs12541643	ABCB11, INS	chr 2:169779448-169887832, chr 11:2181009-2182571	rs853777, rs3842756
Thiazolidinediones	ABCB11, CYP1A2, DMPK, GPX1, IARS1, IGF1R, INHBE, INS, MAPRE3, NR1H3, PDIA5, PPM1G, REEP3, SKAP1, SLC2A1, TP53INP1, WFS1	chr 2:169779448-169887832, chr 15:75041185-75048543, chr 19:46272975-46285810, chr 3:49394609-49396033, chr 9: 94,972,489-95,056,038, chr 15:99192200-99507759, chr 12:57846106-57853063, chr 11:2181009-2182571, chr 2:27193480-27250064, chr 11:47269851-47290396, chr 3:122785909-122944074, chr 2:27604061-27632554, chr 10:65281123-65384883, chr 17:46210802-46507637, chr 1:43391052-43424530, chr 8:95938200-95961639, chr 4:6271576-6304992	rs557462, rs2960192, rs2070737, rs3811699, rs12353096, rs6598541, rs3741414, rs3842754, rs13020526, rs11039154, rs28661248, rs1083864, rs6479911, rs16954324, rs3729548, rs896854, rs6446479	ABCB11, AGER, ALDH8A1, APOC2, CCND2, CDH1, DPAGT1, IDE, INS, NR1H3, PDIA5, PHETA1^a^, PKLR, PLD1, PPIL6, SLC25A20, SLC25A26, SREBF1, TBL2, TF, TMEM86B, TMPRSS6	chr 2:169779448-169887832, chr 6:32148745-32152101, chr 6:135238528-135271260, chr 19:45449243-45452822, chr 12:4382938-4414516, chr 16:68771128-68869451, chr 11:118967213-118979041, chr 10:94211441-94333833, chr 11:2181009-2182571, chr 11:47269851-47290396, chr 3:122785909-122944074, chr 12:111788483-111816899, chr 1:155259630-155271225, chr 3:171318195-171528740, chr 6:109711418-109762374, chr 3:48894369-48936426, chr 3:66119285-66438540, chr 17:17713713-17740325, chr 7:72983262-72993121, chr 3:133464800-133497850, chr 19:55738007-55741647, chr 22:37461476-37505603	rs853777, rs3130349, rs7749106, rs7257476, rs76895963, rs7198799, rs617948, rs11187019, rs3842756, rs11039154, rs28661248, rs874286, rs12067675, rs4894769, rs61318425, rs11708022, rs7630745, rs9894257, rs13232120, rs3811658, rs28678477, rs4820268

Drug class is referred to as ‘Drug’ in the Figures for brevity.

^a^https://www.genecards.org/cgi-bin/carddisp.pl?gene=PHETA1.

### Negative and positive controls

**Fig. 1:**
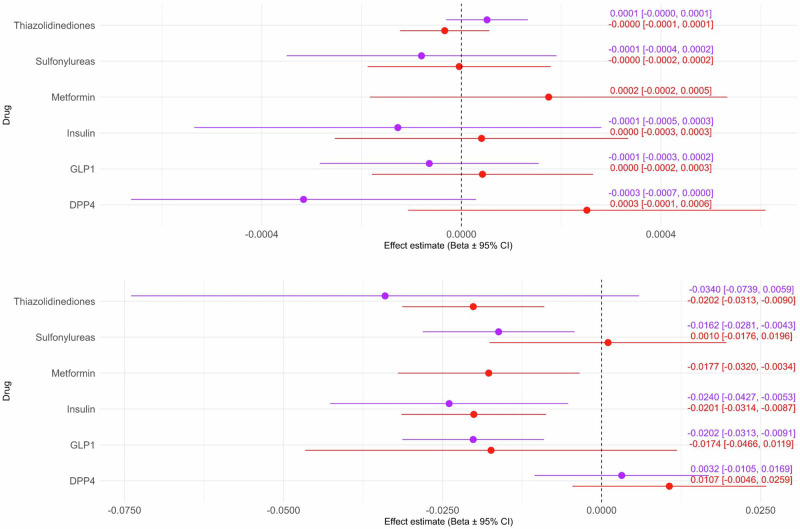
Negative and positive control. Effect estimates refer to the outcome effect estimate (childhood asthma in panel **A** on top, and type 2 diabetes in Panel **B** on the bottom), not the effect estimates coming from the downstream biomarkers. As we were proxying antidiabetic drugs effect, the IVs were coded so that they decrease (negative effect) the two downstream traits (red: fasting glucose, purple: glycated hemoglobin). In successful negative control (panel **A**), we hypothesized that the outcome is not associated with the IVs. In a successful positive control (panel **B**) where the outcome is type 2 diabetes, the outcome effect sizes show negative effect sizes for type 2 diabetes. Note: drug class is referred to as ‘Drug’ in the Figures for brevity.

All IVs passed the negative control, as there were no statistically significant associations between selected IVs and childhood asthma (Fig. 1). Based on our results, the hypothesis of no association is confirmed by the lack of association found in the negative control, and thereby in line with MR assumptions. Checking for directional consistency for decreasing risk of T2D showed that IVs for thiazolidinediones, metformin (FG arm only), insulin/insulin analogues and GLP-1 receptor agonists, as well as IVs in the HbA1c arm for sulfonylureas, passed the positive control (Fig. 1). IVs for Sulfonylureas in the FG arm were a borderline case but were kept as IVs. IVs for DPP-4 inhibitors did not pass the positive control, neither in the FG nor the HbA1c arm, meaning that DPP-4 MR results do not have the same validity as the rest of the drugs. Therefore, DPP-4 F statistics were moved to be reported separately (Supplementary Table 2).

### MR analyses

**Fig. 2:**
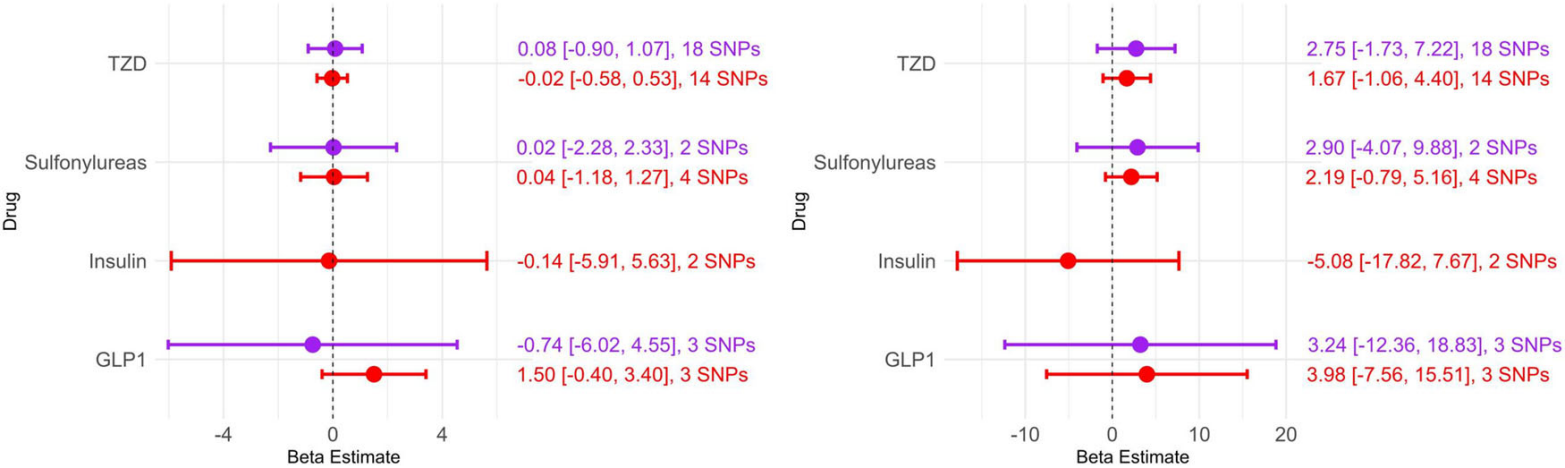
Inverse variance weighted Mendelian randomization analyses per downstream biomarker arm (red: fasting glucose, purple: glycated hemoglobin) using meta-analysis of COURAGE-PD and IPDGC Parkinson’s disease risk (panel **A**, left) and meta-analysis of COURAGE-PD and IPDGC Parkinson’s disease age at onset (panel **B**, right) outcome data. Effect estimates are log odds ratios. Note: TZD stands for thiazolidinediones, and drug class is referred to as ‘Drug’ in the Figures for brevity.

Meta-analyzing the IPDGC and COURAGE data, the main MR analysis (inverse variance weighted, IVW) showed no statistically significant associations between genetic variation in targets of thiazolidinediones, sulfonylureas, insulin/insulin analogues, GLP-1 receptor agonists and PD risk or AAO (Fig. 2, Supplementary Table 3). However, based on the Wald ratio model, the single IV for metformin in the FG arm (rs6598541 in the *IGF1R* gene) was significantly associated with lower PD risk (meta-analysis: B = −2.792, SE = 1.285, *p* = 0.0298) (Supplementary Table 3). Notably, though, this statistical significance disappeared after Bonferroni correction due to testing multiple drug classes. The results remained non-significant in consortium-stratified analyses, except for the association between the single IV for metformin and PD risk in the FG arm in COURAGE-PD prior to Bonferroni adjustment (COURAGE-PD only: B = −5.155, SE = 2.166, *p* = 0.017; IPDGC only: B = −1.509, SE = 1.596, *p* = 0.345) (Supplementary Tables 4, 5). We also found that GLP-1 receptor agonist IVs unadjusted, borderline significantly increased PD risk in the FG arm, in the COURAGE-PD stratification using the IVW model (Beta = 3.024, SE = 1.496, *p* = 0.043). We found no other statistically significant results for any other MR model in any of the consortia (Supplementary Tables 3–5). Although DPP-4 inhibitor IVs failed the positive control, the corresponding non-significant, mixed directionality results are displayed in Supplementary Table 6.

### Sensitivity analyses

There were large variations by different drug classes in statistical uncertainty, likely due to the varying number of IVs available per drug. We plotted post hoc sensitivity analysis scatterplots of MR slopes of the GLP-1 receptor agonists corresponding IVs, to compare MR Egger with the other models, as MR Egger results seemed different from the other three models. This is likely to be due to the low number of SNPs, and not due to instrument weakness, as confirmed by the F statistics (Supplementary Table 1). We examined the IV effect sizes for each exposure and outcome to ensure the model contained no errors (see Supplementary Fig. 1). We found no evidence of colocalization (Supplementary Table 7), in line with the MR results. Leave-one-out MR analysis on the thiazolidinediones IVs were in line with our main MR findings, thus confirming the non-significant results (Supplementary Table 8).

## Discussion

To our knowledge, our drug target MR study used one of the largest GWAS samples both for PD risk and AAO by combining data from the COURAGE-PD and IPDGC consortia, resulting in 42 083 cases/457 090 controls for PD risk and 37 103 cases for PD onset. We found no evidence that genetic variation in antidiabetic drug targets reduces the risk of PD or delays its onset. This project was the first to address this question using GWAS summary statistics from the COURAGE-PD consortium. Moreover, our IV selection approach was novel, since we combined elements and publicly available resources in a unique and comprehensive way by including both the downstream biomarker and eQTL levels. While most previous studies relied on the UK Biobank^24,26,27^ for GWAS data on downstream biomarkers, we used data from a different study population (MAGIC)^33^.

Our results were in line with findings of two previous antidiabetic drug target MR studies^24,27^, but did not support others that found significant decrease^22,26^ in PD risk. Based on the IPDGC datasets, Wang et al. found no significant associations between genetic variation in antidiabetic drug targets and PD risk, AAO or PD progression^27^. We extended these findings with additional data from the COURAGE-PD consortium^28,29^. A difference between our study and Wang et al. was the IV selection process, as those authors used GWAS summary statistics on medication-use from the UK Biobank^34,35^, whereas we took a biologically informed approach based on biomarkers and eQTL. The other study supporting our findings of a lack of association between genetically predicted antidiabetic drug effects and PD risk or AAO^24^ was based on GWAS summary statistics from the FinnGen biobank and relied on IVs identified by previous studies^11,36^. Some of these IVs were selected using downstream biomarkers^11^, and similar to our approach, the metformin IVs were selected through a combination of biomarkers and eQTL^36^. Yet, those authors identified metformin-related drug targets in different genes than we did^36^. A potential explanation for this is that the mechanism of action of metformin is complex and not completely understood^37^.

Our findings did not confirm the drug target MR findings of Storm et al., who found preliminarily positive evidence for repurposing metformin for PD prevention using the IPDGC datasets^22^. Storm et al. selected IVs through prefrontal cortex and blood tissue-specific eQTL and pQTL filtering of druggable genes but did not filter specifically for diabetes-related traits^22^. Further, our findings did not corroborate a more recent drug target MR study that reported evidence for repurposing sulfonylureas to prevent PD based on the IPDGC dataset^26^ with IVs selection in a similar fashion to Zhao et al.^24^, but using IVs from a study on Alzheimer’s disease selected from the UK Biobank^11^. It is important to note though, that using the same sulfonylureas IVs, these results were not reproduced in the Finngen PD risk dataset^24^. As we were unable to instrument genetic variation in targets of SGLT2 inhibitors, a direct comparison with a previous drug target MR study that reported increased PD risk related to SGLT2 inhibitors^25^ is precluded.

We found a slight signal for decreased PD risk related to a SNP in the *IGF1R* gene (Insulin-like growth factor 1 receptor), a drug target of metformin, in the FG arm before Bonferroni adjustment in COURAGE-PD and the meta-analysis. The variant has been implicated in inflammatory processes in gout^38^, and is expressed in the heart and to a lesser extent in several other tissues, including the brain (amygdala)^39^. Several lines of evidence support the relevance of IGF-1/IGF1R in the pathophysiology and development of PD^40^.

Notably, there was very little overlap between IVs included in previous drug target MR studies on antidiabetics and PD risk and our study, which is likely explained by the different study populations of the GWAS data for downstream biomarkers, and our different approach. Only one IV in our study (rs76895963) in the *CCND2* (Cyclin D2 gene) was included in a previous study^27^. However, two of the drug target genes in our study, *PPIL6* (Peptidylprolyl Isomerase Like 6) and *PHETA1* (PH Domain Containing Endocytic Trafficking Adaptor 1), were also GWAS hits in the COURAGE-PD risk GWAS^30^. There was no overlap between the hits identified by one of the latest GWASes on PD risk^41^ and our IVs, but interestingly there was one overlapping gene: *SREBF1*, which was a novel GWAS locus corresponding to GLP-1 receptor agonists and thiazolidinediones in the HbA1c arm among our IVS.

Observational studies on antidiabetics and PD risk have reported mixed results. A meta-analysis of population-based cohort studies found decreased risk of neurodegenerative diseases related to metformin, but no association with PD^13^. SGLT2 inhibitors have shown some potential for repurposing in a population-based Korean cohort study^42^. Thiazolidinediones were associated with lower PD risk in a population-based retrospective cohort study of T2D patients in China^43^. A meta-analysis of observational evidence from Taiwan and the UK found that pioglitazone (belonging to the thiazolidinediones drug class) was linked to decreased PD risk in a dose-response manner^44^, but another meta-analysis reported no association between certain thiazolidinediones, metformin, sulfonylureas, DPP-4 inhibitors and PD risk^45^. A major difference between drug target MR and observational studies is the duration of exposure. Drug target MR studies proxy lifelong exposure to the drug of interest, while observational studies usually investigate much shorter periods^20^. Additionally, observational studies are limited by different biases, such as confounding and reverse causation, which are minimized in the MR approach.

Large-scale observational data are limited to participants with diabetes. A meta-analysis of clinical trials for repurposing newer antidiabetic drugs, also including persons with heart failure and chronic kidney disease, found decreased risk of PD related to SGLT2 inhibitors but was limited by small numbers, short follow-up and lack of exclusion of PD cases at baseline^46^. Although early clinical trials of GLP-1 receptor agonists showed promising results for disease modification in PD^47^, some later results showed limited clinical relevance^48^, and a recent phase 3 trial of the GLP-1 receptor agonist exenatide for disease modification was negative^49^.

One of the main strengths of this study was the combination of data from the COURAGE-PD and IPDGC consortia, which to our knowledge, resulted in the largest European-ancestry GWAS samples both for PD risk and AAO. Additionally, we also used data from a large-scale consortium (MAGIC) for the IV selection, in contrast to most other similar studies that relied on the UK Biobank^24,26,27^. To ensure biological relevance in the IV selection process, i.e. that the SNP influences gene expression, we implemented an eQTL-filtering step^20,36,50^. Furthermore, to triangulate findings, we used sensitivity analyses, including positive and negative controls, in line with current guidelines for performing and reporting drug target MR studies^51^, also displayed in Supplementary Fig. 2.

However, there are limitations to our study. First, we were unable to instrument SGLT2 inhibitors. Second, the outcome GWAS summary statistics were mostly based on persons of European ancestry, resulting in generalizability mainly to European ancestry populations. Further research on different populations is needed, especially considering that much of the observational evidence in favor of repurposing antidiabetic drugs for PD come from East Asia. Finally, it is important to acknowledge the general limitation stemming from the study design that despite our best efforts to follow best practice and conduct sensitivity analyses, we cannot fully exclude that there is pleiotropy at play.

In conclusion, using a drug target MR framework with a comprehensive approach to IV selection and summary statistics from a large PD GWAS sample, we found no significant association between genetic variation in antidiabetic drug targets and PD risk or AAO. These results suggest that there is no indication for repurposing any of the examined antidiabetic drugs for primary prevention or delayed onset of PD. Future research should focus on investigating other drug classes as potential strategies for PD prevention.

## Methods

### Study design: drug target MR

We used a drug target MR framework to investigate whether genetic variation in antidiabetic drug targets would lower the risk or delay the onset of PD.

MR is an analytic approach in which environmental exposure is proxied through genetic variants, used as instrumental variables (IVs)^20,21^. In contrast to conventional observational designs, MR helps to overcome issues such as unresolved confounding and reverse causation thanks to Mendel’s law of independent assortment, meaning that the inheritance of genetic variants related to the trait of interest is independent of (i.e. randomized with respect to) the inheritance of other traits^21^. This makes an MR study similar to a randomized controlled trial, enabling causal inference if certain assumptions are met^52^.

Drug target MR is a specific type of MR concerned with the validation of drug targets, often with the aim to explore drug repurposing possibilities^20^. Unlike conventional MR, which typically uses genetic variants (IVs) associated with an exposure, drug target MR restricts instruments to cis-acting variants located near or within the gene encoding the drug target. IVs should pass three assumptions in a drug target MR study^20^: (i) *relevance assumption*, i.e. the drug affects the relevant tissues, e.g. by incorporating downstream biomarkers in IV filtering; (ii) *independence assumption*, the relationship between IVs and the outcome are independent of confounders; (iii) *exclusion restriction* assumption (or 'no horizontal pleiotropy' assumption^53^), effects from pleiotropic mechanisms should be separate from the drug target’s effect^20^. The first assumption is testable; the remaining two are not testable, but falsifiable^54^. By filtering variants within the cis-regions of relevant genes, including an eQTL step in the IV selection process, incorporating GWASes on downstream biomarkers, such as FG and HbA1c, and applying LD clumping as the final step alongside downstream biomarkers, we aimed to ensure biological relevance fulfilling the three assumptions.

### Data resources

Genes linked to the antidiabetic drugs under study were identified from PubChem^55^ and Drug-Gene Interaction Database (Beta version)^56^. All drugs are listed in Supplementary Table 9.

To proxy antidiabetic drug effects, we used summary statistics from two GWASes from the Meta-Analyses of Glucose and Insulin-related traits Consortium (MAGIC): one on fasting glucose (FG) and another on glycated hemoglobin (HbA1c)^33,57^. Both datasets were restricted to European populations to harmonize with the available outcome datasets. To our knowledge, the MAGIC samples do not overlap with the outcome GWASes. For the outcomes of PD risk and age at onset (AAO), we relied on GWAS summary statistics from two consortiums for each: IPDGC^31,32^ and the COURAGE-PD consortium^28–30^. As there is some sample overlap between the IPDGC and COURAGE-PD, we excluded the part of the sample from COURAGE-PD that overlaps with IPDGC^41–44^. The PD risk datasets included cases and PD-free controls, while the AAO GWAS datasets only focused on PD cases with the aim to identify genetic loci associated with the variation in onset age. For our meta-analyses of these two GWASes, our sample included 42 083 cases/457 090 controls for risk and 37 103 PD cases for AAO, thus resulting in the best-powered study so far.

We also used multi-tissue eQTL data, selected from The Genotype-Tissue Expression (GTEx) Portal^39,58^. Sample size and further details on the GWASes used and the eQTL dataset can be found in Supplementary Table 10.

### Selection of IVs

**Fig. 3:**
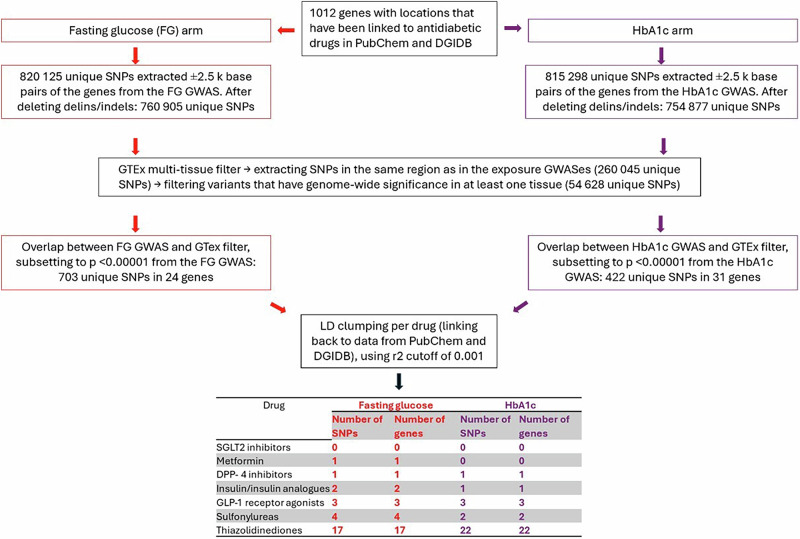
Flowchart of instrumental variable selection. HbA1c stands for glycated hemoglobin, and drug class is referred to as ‘Drug’ in the Figures for brevity.

The IV selection process is described in Fig. 3. We first identified a list of antidiabetic drugs for the drug classes of interest (e.g. tolazamide for sulfonylureas, exenatide for GLP-1 receptor agonists, etc., see Supplementary Table 9) and genes associated with them. We defined our IVs as SNPs. IV selection was divided into two parallel, downstream biomarker arms, each relating to diabetes: FG and HbA1c, corresponding to the proxied antidiabetic drug effects (lowering FG and HbA1c, respectively). The two arms were kept separate to facilitate interpretation of the results. SNPs were extracted from these two GWASes on glycemic traits in the cis-variant region of the genes (defined as ±2500 base pairs of the gene location, similar to previous research^11^), as well as from within the gene region. To ensure biological relevance, SNPs were then filtered requiring genome-wide significance (*p* < 5 ×10⁻⁸) in at least one of 48 tissues in the GTEx multi-tissue eQTL resource^39,59^, since most antidiabetic drug targets are expressed across multiple tissues. We then filtered further by requiring a strong association with FG and HbA1c (*p* < 0.00001 in their respective GWASes). SGLT2 inhibitor IV candidates were lost during the latter filtering step, as they had higher *p* values. We used both eQTL and GWASes on downstream biomarkers to strengthen the plausibility of the relevance- and ‘no horizontal pleiotropy’ assumptions in the MR design^53^, since having both filters helps with proxying the protein quantitative trait loci (pQTL) level^20,36^. We then performed LD clumping of the SNPs per drug class separately in each downstream biomarker arm, retaining SNPs using the r^2^ cutoff of 0.001; this LD clumping step contributed towards the independence assumption of MR IV selection^20^. The remaining SNPs were then linked back to genes, and then to drugs whose effects the genes proxy, and finally to the overall drug classes. To ensure that the SNPs proxied antidiabetic drug effects consistently and to allow interpretation of the positive and negative control as well as MR analyses, SNPs were coded so that effect alleles lower FG and HbA1c levels, respectively. F statistics were calculated to test the strength of each IV set.

### Positive and negative control

To ascertain directional consistency of genetically predicted drug effects with clinical trial evidence/drug mechanisms, we performed positive and negative control analyses for the selected IVs per drug class^11^ by random effects meta-analysis of individual SNP effect sizes on the control outcome.

For the positive control, we used T2D as the outcome and corresponding GWAS summary statistics from FinnGen (T2D, wide definition)^60^. As the SNPs were coded so that the effect allele lowers FG and HbA1c, we expected that they would also be associated with lower risk for T2D. Therefore, drug classes with pooled estimates showing directional consistency were considered to pass the positive control.

For the negative control, childhood asthma risk was chosen as the outcome, as we hypothesized that the selected exposures and drug targets are not causally affecting childhood asthma risk. We used GWAS summary statistics on childhood asthma (age <16) from the UK Biobank through the IEU OpenGWAS project^61^. In line with our hypothesis of no relationship between our IVs and risk for childhood asthma, drug classes that pass the negative control should have a pooled estimate showing no significant effect.

### MR analyses

For the MR analyses, our outcome GWAS summary statistics were PD risk and PD AAO. We used 5 different MR models (Wald ratio, inverse variance weighted [IVW], MR Egger, weighted mean and weighted median) and conducted analyses for each outcome by meta-analyzing SNPs from the two consortia using fixed-effects models, plus analyses stratified by consortium. The analyses were kept separate depending on the downstream biomarker used (FG or HbA1c). LD clumping and MR analyses were carried out using the TwoSampleMR package in R (version 0.6.14)^62^.

All analyses were conducted in R (versions 4.2.3 and R 4.4.0)^63^. Genomic locations corresponded to the GRCH37/hg19 genome assembly via the R/Bioconductor package biomaRt^64,65^.

### Sensitivity analyses

To ensure the validity of our results, we conducted sensitivity analyses. These included post-analysis scatterplots to investigate effect sizes, confidence intervals and MR model slopes further where we deemed results as unusual or extreme. We have also conducted colocalization analysis where MR signals were the most pronounced, to be able to answer the question whether the exposure signal and the outcome signal are truly driven by the same variant or whether it only appears so due to LD^66^. Since thiazolidinediones had the highest number of IVs, leave-one-out MR using IVW was also conducted to investigate whether there are any outliers.

## Supplementary information

Supplementary information

## Data Availability

Apart from COURAGE-PD datasets, all data used are publicly available. For details, see Supplementary Table 10.

## Code availability

R codes are available on Zenodo (10.5281/zenodo.19388329), apart from the codes working directly on COURAGE-PD data.

## References

[1] Steinmetz, J. D. et al. Global, regional, and national burden of disorders affecting the nervous system, 1990–2021: a systematic analysis for the Global Burden of Disease Study 2021. *Lancet Neurol.* **23**, 344–381 (2024). 38493795 10.1016/S1474-4422(24)00038-3 PMC10949203

[2] Bloem, B. R., Okun, M. S. & Klein, C. Parkinson’s disease. *Lancet* pp. 2284–2303, https://linkinghub.elsevier.com/retrieve/pii/S014067362100218X (2021). 10.1016/S0140-6736(21)00218-X 33848468

[3] Kafantari, E., Atterling Brolin, K., Wallenius, J., Swanberg, M. & Puschmann, A. WES-based screening of a Swedish patient series with Parkinson’s disease. *Genes* **16**, 1482 (2025). 41465155 10.3390/genes16121482 PMC12732595

[4] Santiago, J. A. & Potashkin, J. A. Integrative network analysis unveils convergent molecular pathways in Parkinson’s disease and diabetes. *PLoS One* **8**(12), e83940 (2013). 24376773 10.1371/journal.pone.0083940 PMC3869818

[5] Szendroedi, J., Phielix, E. & Roden, M. The role of mitochondria in insulin resistance and type 2 diabetes mellitus. *Nat. Rev. Endocrinol.* **8**, 92–103 (2011). 21912398 10.1038/nrendo.2011.138

[6] Cheong, J. L. Y., de Pablo-Fernandez, E., Foltynie, T. & Noyce, A. J. The association between Type 2 Diabetes Mellitus and Parkinson’s disease. *J. Parkinsons Dis.* **10**, 775–789 (2020). 32333549 10.3233/JPD-191900 PMC7458510

[7] Athauda, D. & Foltynie, T. Insulin resistance and Parkinson’s disease: a new target for disease modification?. *Prog. Neurobiol.* **145–146**, 98–120 (2016). 27713036 10.1016/j.pneurobio.2016.10.001

[8] Aguirre-Vidal, Y., Montes, S., Mota-López, A. C. & Navarrete-Vázquez, G. Antidiabetic drugs in Parkinson’s disease. *Clin. Park Relat. Disord.* **11**, 100265 (2024). 39149559 10.1016/j.prdoa.2024.100265 PMC11325349

[9] Pushpakom, S. et al. Drug repurposing: progress, challenges and recommendations. *Nat. Rev. Drug Discov.* **18**, 41–58 (2018). 30310233 10.1038/nrd.2018.168

[10] Zhu, S., Bai, Q., Li, L. & Xu, T. Drug repositioning in drug discovery of T2DM and repositioning potential of antidiabetic agents. *Comput Struct. Biotechnol. J.* **20**, 2839 (2022). 35765655 10.1016/j.csbj.2022.05.057 PMC9189996

[11] Tang, B. et al. Genetic variation in targets of antidiabetic drugs and alzheimer disease risk: a Mendelian randomization study. *Neurology* **99**, E650–E659 (2022). 35654594 10.1212/WNL.0000000000200771 PMC9484609

[12] Birajdar, S. V., Mazahir, F., Alam, M. I., Kumar, A. & Yadav, A. K. Repurposing and clinical attributes of antidiabetic drugs for the treatment of neurodegenerative disorders. *Eur. J. Pharm.* **961**, 176117 (2023). 10.1016/j.ejphar.2023.176117 37907134

[13] Zhang, Y. et al. Metformin and the risk of neurodegenerative diseases in patients with diabetes: a meta-analysis of population-based cohort studies. *Diabet. Med.* **39**, e14821 (2022). 35213749 10.1111/dme.14821

[14] Shi, Q., Liu, S., Fonseca, V. A., Thethi, T. K. & Shi, L. Effect of metformin on neurodegenerative disease among elderly adult US veterans with type 2 diabetes mellitus. *BMJ Open* **9**10.1136/BMJOPEN-2018-024954 (2019). 10.1136/bmjopen-2018-024954 PMC6677947 31366635

[15] Wahlqvist, M. L. et al. Metformin-inclusive sulfonylurea therapy reduces the risk of Parkinson’s disease occurring with Type 2 diabetes in a Taiwanese population cohort. *Parkinsonism Relat. Disord.* **18**, 753–758 (2012). 22498320 10.1016/j.parkreldis.2012.03.010

[16] Brauer, R. et al. Diabetes medications and risk of Parkinson’s disease: a cohort study of patients with diabetes. *Brain* **143**, 3067–3076 (2020). 33011770 10.1093/brain/awaa262 PMC7794498

[17] McFarthing, K. et al. Parkinson’s disease drug therapies in the clinical trial pipeline: 2024 update. *J. Parkinsons Dis.* **14**, 899–912 (2024). 39031388 10.3233/JPD-240272 PMC11307066

[18] Sunnarborg, K. et al. Association between different diabetes medication classes and risk of Parkinson’s disease in people with diabetes. *Pharmacoepidemiol Drug Saf.* **31**, 875 (2022). 35505634 10.1002/pds.5448 PMC9542001

[19] Lin, K. J. et al. Two birds one stone: the neuroprotective effect of antidiabetic agents on Parkinson disease—focus on sodium-glucose cotransporter 2 (SGLT2) inhibitors. *Antioxidants* **10**, 10.3390/ANTIOX10121935 (2021). 10.3390/antiox10121935 PMC8750793 34943038

[20] Daghlas, I. & Gill, D. Mendelian randomization as a tool to inform drug development using human genetics. *Camb. Prisms Precis. Med.* **1**, e16 (2023). 10.1017/pcm.2023.5 PMC10953771 38550933

[21] Ebrahim, S. & Davey Smith, G. Mendelian randomization: can genetic epidemiology help redress the failures of observational epidemiology?. *Hum. Genet.* **123**, 15–33 (2008). 18038153 10.1007/s00439-007-0448-6

[22] Storm, C. S. et al. Finding genetically-supported drug targets for Parkinson’s disease using Mendelian randomization of the druggable genome. *Nat. Commun.* **12**, 1–14 (2021). 34930919 10.1038/s41467-021-26280-1 PMC8688480

[23] Mor, D. E. et al. Metformin rescues Parkinson’s disease phenotypes caused by hyperactive mitochondria. *Proc. Natl. Acad. Sci. USA* **117**, 26438–26447 (2020). 33024014 10.1073/pnas.2009838117 PMC7585014

[24] Zhao, Y., Fei, L. & Duan, Y. Movement disorders related to antidiabetic medications: a real-world pharmacovigilance study. *Prog. Neuropsychopharmacol. Biol. Psychiatry* **135**, 111128 (2024). 39181309 10.1016/j.pnpbp.2024.111128

[25] Liu, J., Shi, X. & Shao, Y. Sodium-glucose cotransporter 1/2 inhibition and risk of neurodegenerative disorders: a Mendelian randomization study. *Brain Behav.* **14**, e3624 (2024). 39010704 10.1002/brb3.3624 PMC11250420

[26] Aziz, N. A. & Wüllner, U. Genetic variants in sulfonylurea targets affect Parkinson’s disease risk: a two-sample Mendelian randomization study. *Mov. Disord.* **38**, 703–705 (2023). 36782054 10.1002/mds.29341

[27] Wang, Q. et al. Identifying potential repurposable medications for Parkinson’s disease through Mendelian randomization analysis. *Sci. Rep.* **14**, 1–11 (2024). 39181920 10.1038/s41598-024-70758-z PMC11344818

[28] Domenighetti, C. et al. Association of Body Mass Index and Parkinson Disease A Bidirectional Mendelian Randomization Study. *Neurology* **10**, 103 (2024). 10.1212/WNL.0000000000209620 PMC11759940 38986057

[29] Grover, S. et al. Genome-wide association and meta-analysis of age at onset in Parkinson disease: evidence from the COURAGE-PD Consortium. *Neurology* **99**, E698–E710 (2022). 35970579 10.1212/WNL.0000000000200699 PMC9484604

[30] Landoulsi. Z. et al. Genome-wide association study of copy number variations in Parkinson’s disease. *medRxiv* https://www.medrxiv.org/content/10.1101/2024.08.21.24311915v2.full-text (2025). 10.1038/s41531-025-01245-z PMC13323976 42009659

[31] Blauwendraat, C. et al. Parkinson’s disease age at onset genome-wide association study: defining heritability, genetic loci, and α-synuclein mechanisms. *Mov. Disord.* **34**, 866–75 (2019). 30957308 10.1002/mds.27659 PMC6579628

[32] Nalls, M. A. et al. Identification of novel risk loci, causal insights, and heritable risk for Parkinson’s disease: a meta-analysis of genome-wide association studies. *Lancet Neurol.* **18**, 1091–102 (2019). 31701892 10.1016/S1474-4422(19)30320-5 PMC8422160

[33] Magic Investigators - Data download. https://magicinvestigators.org/downloads/ (2025).

[34] UK Biobank - UK Biobank. https://www.ukbiobank.ac.uk/ (2025).

[35] Wu, Y. et al. Genome-wide association study of medication-use and associated disease in the UK Biobank. *Nat. Commun.* **10**, 1–10 (2019). 31015401 10.1038/s41467-019-09572-5 PMC6478889

[36] Zheng, J. et al. Efficacy of metformin targets on cardiometabolic health in the general population and non-diabetic individuals: a Mendelian randomization study. *EBioMedicine* **96**, 104803 (2023). 37734206 10.1016/j.ebiom.2023.104803 PMC10514430

[37] Foretz, M., Guigas, B. & Viollet, B. Metformin: update on mechanisms of action and repurposing potential. *Nat. Rev. Endocrinol.* **19**, 460–76 (2023). 37130947 10.1038/s41574-023-00833-4 PMC10153049

[38] Gaal, O. I. et al. GWAS-identified hyperuricemia-associated IGF1R variant rs6598541 has a limited role in urate mediated inflammation in human mononuclear cells. *Sci. Rep.* **14**, 3565 (2024). 38347000 10.1038/s41598-024-53209-7 PMC10861580

[39] GTEx Portal. https://www.gtexportal.org/home/downloads/adult-gtex/qtl (2025).

[40] Castilla-Cortázar, I., Aguirre, G. A., Femat-Roldán, G., Martín-Estal, I. & Espinosa, L. Is insulin-like growth factor-1 involved in Parkinson’s disease development? *J. Transl. Med.* **18**, 1–17 (2020). 32046737 10.1186/s12967-020-02223-0 PMC7014772

[41] The Global Parkinson’s Genetics Program (GP2) & Leonard, H. L. Novel Parkinson’s disease genetic risk factors within and across European populations. *medRxiv,*10.1101/2025.03.14.24319455 (2025).

[42] Kim, H. K. et al. SGLT2 inhibitor use and risk of dementia and Parkinson disease among patients with type 2 diabetes. *Neurology* **103**, e209805 (2024). 39292986 10.1212/WNL.0000000000209805

[43] Zhao, H. et al. Thiazolidinedione use and risk of Parkinson’s disease in patients with type 2 diabetes mellitus. *NPJ Parkinsons Dis.* **8**, 1–9 (2022). Dec 1. 36271052 10.1038/s41531-022-00406-8 PMC9587207

[44] Chen, L., Tao, Y., Li, J. & Kang, M. Pioglitazone use is associated with reduced risk of Parkinson’s disease in patients with diabetes: a systematic review and meta-analysis. *J. Clin. Neurosci.* **106**, 154–8 (2022). 36335768 10.1016/j.jocn.2022.10.023

[45] Qin, X. et al. Association between diabetes medications and the risk of Parkinson’s disease: a systematic review and meta-analysis. *Front. Neurol.* **12**, 678649 (2021). 34349721 10.3389/fneur.2021.678649 PMC8326375

[46] Tang, H. et al. Meta-analysis of association between newer glucose-lowering drugs and risk of Parkinson’s disease. *Mov. Disord. Clin. Pr.* **10**, 1659–65 (2023). 10.1002/mdc3.13893 PMC10654811 37982117

[47] Athauda, D. et al. Exenatide once weekly versus placebo in Parkinson’s disease: a randomised, double-blind, placebo-controlled trial. *Lancet* **390**, 1664–75 (2017). 28781108 10.1016/S0140-6736(17)31585-4 PMC5831666

[48] Meissner, W. G. et al. Trial of lixisenatide in early Parkinson’s disease. *N. Engl. J. Med.* **390**, 1176–85 (2024). 38598572 10.1056/NEJMoa2312323

[49] Vijiaratnam, N. et al. Exenatide once a week versus placebo as a potential disease-modifying treatment for people with Parkinson’s disease in the UK: a phase 3, multicentre, double-blind, parallel-group, randomised, placebo-controlled trial. *Lancet* **405**, 627–36 (2025). 39919773 10.1016/S0140-6736(24)02808-3

[50] Zheng, G., Chattopadhyay, S., Sundquist, J., Sundquist, K. & Ji, J. Antihypertensive drug targets and breast cancer risk: a two-sample Mendelian randomization study. *Eur. J. Epidemiol.* **39**, 535–48 (2024). 38396187 10.1007/s10654-024-01103-x PMC11219410

[51] Burgess, S. et al. Guidelines for performing Mendelian randomization investigations: update for summer 2023. *Wellcome Open Res.* **4**, 186 (2023). 32760811 10.12688/wellcomeopenres.15555.1 PMC7384151

[52] Gill, D., Walker, V. M., Martin, R. M., Davies, N. M. & Tzoulaki, I. Comparison with randomized controlled trials as a strategy for evaluating instruments in Mendelian randomization. *Int J. Epidemiol.* **49**, 1404–6 (2020). 31764983 10.1093/ije/dyz236

[53] Schmidt, A. F. et al. Genetic drug target validation using Mendelian randomisation. *Nat. Commun.* **11**, 1–12 (2020). 32591531 10.1038/s41467-020-16969-0 PMC7320010

[54] Gill, D. et al. Mendelian randomization for studying the effects of perturbing drug targets [version 1; peer review: awaiting peer review]. *Wellcome Open Res* **6**, 1–11 (2021). 33644404 10.12688/wellcomeopenres.16544.1 PMC7903200

[55] PubChem. https://pubchem.ncbi.nlm.nih.gov/ (2025).

[56] DGIdb. https://beta.dgidb.org/ (2025).

[57] Chen, J. et al. The trans-ancestral genomic architecture of glycemic traits. *Nat. Genet.* **53**, 840–60 (2021). 34059833 10.1038/s41588-021-00852-9 PMC7610958

[58] Lonsdale, J. et al. The Genotype-Tissue Expression (GTEx) project. *Nat. Genet* **45**, 580–5 (2013). 23715323 10.1038/ng.2653 PMC4010069

[59] Quiver, M. H. & Lachance, J. Adaptive eQTLs reveal the evolutionary impacts of pleiotropy and tissue-specificity while contributing to health and disease. *Hum. Genet. Genomics Adv.* **3**, 100083 (2021). 10.1016/j.xhgg.2021.100083 PMC8756519 35047867

[60] Kurki, M. I. et al. FinnGen provides genetic insights from a well-phenotyped isolated population. *Nature* **613**, 508–18 (2023). 36653562 10.1038/s41586-022-05473-8 PMC9849126

[61] Trait: Childhood asthma (age<16) - IEU OpenGWAS project. https://gwas.mrcieu.ac.uk/datasets/ukb-d-ASTHMA_CHILD/ (2025).

[62] Hemani, G. et al. The MR-Base platform supports systematic causal inference across the human phenome. *eLife* **7**, e34408 (2018). 2018. 29846171 10.7554/eLife.34408 PMC5976434

[63] R Core Team. *R: A Language and Environment for Statistical Computing* (R Foundation for Statistical Computing, 2023).

[64] Durinck, S. et al. BioMart and Bioconductor: a powerful link between biological databases and microarray data analysis. *Bioinformatics* **21**, 3439–40 (2005). 16082012 10.1093/bioinformatics/bti525

[65] Durinck, S., Spellman, P. T., Birney, E. & Huber, W. Mapping identifiers for the integration of genomic datasets with the R/Bioconductor package biomaRt. *Nat. Protoc.* **4**, 1184–91 (2009). 19617889 10.1038/nprot.2009.97 PMC3159387

[66] Giambartolomei, C. et al. Bayesian test for colocalisation between pairs of genetic association studies using summary statistics. *PLoS Genet* **10**, e1004383 (2014). 24830394 10.1371/journal.pgen.1004383 PMC4022491

