# Supplementary material for: Genetic variation in antidiabetic drug targets: associations with Parkinson’s disease risk and age at onset
Source: NPJ Parkinsons Dis. 2026 May 28;12:127. doi: 10.1038/s41531-026-01398-5 (PMC13219586; doi:10.1038/s41531-026-01398-5)
Supplement: Supplementary file 1 — Supplementary information [file 41531_2026_1398_MOESM1_ESM.pdf]

SUPPLEMENTS

Meta-analysis												
Drug	PD risk						PD AAO					
	Fasting glucose (N =200 622)			HbA1c (N = 146 806)			Fasting glucose (N =200 622)			HbA1c (N = 146 806)		
	r2	k	F stat	r2	k	F stat	r2	k	F stat	r2	k	F stat
Metformin	0.00024	1	48.071				0.00024	1	48.071			
Insulin	0.00037	2	37.151	0.000174	1	25.506	0.00037	2	37.151	0.000174	1	25.506
GLP1	0.000383	3	25.631	0.000586	3	28.688	0.000383	3	25.631	0.000586	3	28.688
Sulfonylureas	0.004997	4	251.875	0.003622	2	266.822	0.004997	4	251.875	0.003622	2	266.822
Thiazolidinediones	0.007028	14	101.415	0.008179	18	67.251	0.007028	14	101.415	0.008179	18	67.251
COURAGE-PD												
Drug	PD risk						PD AAO					
	Fasting glucose (N =200 622)			HbA1c (N = 146 806)			Fasting glucose (N =200 622)			HbA1c (N = 146 806)		
	r2	k	F stat	r2	k	F stat	r2	k	F stat	r2	k	F stat
Metformin	0.00024	1	48.071				0.00024	1	48.071			
Insulin	0.00037	2	37.151	0.000174	1	25.506	0.00037	2	37.151	0.000174	1	25.506
GLP1	0.000383	3	25.631	0.000586	3	28.688	0.000383	3	25.631	0.000586	3	28.688
Sulfonylureas	0.004997	4	251.875	0.003622	2	266.822	0.004997	4	251.875	0.003622	2	266.822
Thiazolidinediones	0.007028	14	101.415	0.008179	18	67.251	0.007837	17	93.207	0.008641	20	63.97
IPDGC												
Drug	PD risk						PD AAO					
	Fasting glucose (N =200 622)			HbA1c (N = 146 806)			Fasting glucose (N =200 622)			HbA1c (N = 146 806)		
	r2	k	F stat	r2	k	F stat	r2	k	F stat	r2	k	F stat
Metformin	0.00024	1	48.071				0.00024	1	48.071			
Insulin	0.00037	2	37.151	0.000174	1	25.506	0.00037	2	37.151	0.000174	1	25.506
GLP1	0.000383	3	25.631	0.000586	3	28.688	0.000383	3	25.631	0.000586	3	28.688
Sulfonylureas	0.004997	4	251.875	0.003622	2	266.822	0.004997	4	251.875	0.003622	2	266.822
Thiazolidinediones	0.007028	14	101.415	0.008386	19	65.337	0.007028	14	101.415	0.008179	18	67.251

Supplementary table 1. F statistics

	Meta-analysis											
	PD risk						PD AAO					
	Fasting glucose			Hba1c			Fasting glucose			HbA1c		
Drug	R2	K	F stat	R2	K	F stat	R2	K	F stat	R2	K	F stat
DPP-4	0.000126	1	25.354	0.000332	1	48.787	0.000126	1	25.354	0.000332	1	48.787
	COURAGE-PD											
DPP-4	0.000126	1	25.354	0.000332	1	48.787	0.000126	1	25.354	0.000332	1	48.787
	IPDGC											
DPP-4	0.000126	1	25.354	0.000332	1	48.787	0.000126	1	25.354	0.000332	1	48.787

Supplementary table 2. DPP-4 F statistics

Meta-analysis																				
	PD risk										PD AAO									
	Fasting glucose					HbA1c					Fasting glucose					HbA1c				
Drug	MR method	# SNPs	Beta	Standard error	P	MR method	# SNPs	Beta	Standard error	P	MR method	# SNPs	Beta	Standard error	P	MR method	# SNPs	Beta	Standard error	P
Metformin	Wald ratio	1	-2,792	1,285	0,0298						Wald ratio	1	-8,941	8,734	0,306					
Insulin	IVW	2	-0,14	2,945	0,962	Wald ratio	1	4,88	2,232	0,0289	IVW	2	-5,077	6,502	0,435	Wald ratio	1	-1,13	15,226	0,941
GLP1	IVW	3	1,503	0,97	0,121	IVW	3	-0,739	2,696	0,784	IVW	3	3,979	5,885	0,499	IVW	3	3,236	7,956	0,684
GLP1	MR Egger	3	6,508	10,674	0,651	MR Egger	3	26,278	44,532	0,661	MR Egger	3	24,184	66,147	0,777	MR Egger	3	44,075	108,946	0,755
GLP1	Weighted median	3	1,254	1,172	0,285	Weighted median	3	-1,319	1,656	0,426	Weighted median	3	0,737	7,458	0,921	Weighted median	3	1,945	8,862	0,826
GLP1	Weighted mode	3	0,834	1,483	0,63	Weighted mode	3	-1,796	2,06	0,475	Weighted mode	3	-1,763	9,206	0,866	Weighted mode	3	-0,216	10,76	0,986
Sulfonylureas	IVW	4	0,042	0,624	0,947	IVW	2	0,023	1,177	0,985	IVW	4	2,188	1,517	0,149	IVW	2	2,905	3,557	0,414
Sulfonylureas	MR Egger	4	-0,689	0,847	0,502						MR Egger	4	0,349	2,208	0,889					
Sulfonylureas	Weighted median	4	-0,125	0,233	0,592						Weighted median	4	1,948	1,535	0,204					
Sulfonylureas	Weighted mode	4	-0,172	0,224	0,5						Weighted mode	4	1,789	1,599	0,345					
TZD	IVW	14	-0,025	0,284	0,931	IVW	18	0,083	0,504	0,869	IVW	14	1,671	1,393	0,23	IVW	18	2,745	2,282	0,229
TZD	MR Egger	14	-0,141	0,425	0,745	MR Egger	18	-0,615	1,011	0,551	MR Egger	14	2,552	2,056	0,238	MR Egger	18	0,826	4,507	0,857
TZD	Weighted median	14	-0,167	0,228	0,464	Weighted median	18	-0,2	0,453	0,66	Weighted median	14	1,737	1,54	0,259	Weighted median	18	2,732	3,244	0,4
TZD	Weighted mode	14	-0,119	0,244	0,634	Weighted mode	18	-0,186	0,437	0,676	Weighted mode	14	1,809	1,609	0,281	Weighted mode	18	2,996	3,356	0,384

Supplementary table 3. Meta-analysis of IPDGC and COURAGE-PD MR, unadjusted P. Note: IVW = Inverse variance weighted, TZD = Thiazolidinediones

Footnotes: Of the original 17 IVs (SNPs) for TZD in the FG arms, three (rs1083864, rs2070737, rs6446479) were filtered out during harmonisation (due to being palindromic with intermediate allele frequencies). Of the original 22 SNPs for TZD in the HbA1c arms, two (rs13232120, rs4894769) were excluded from the PD risk analysis for similar reasons, one SNP (rs28678477) was missing from the IPDGC and COURAGE-PD risk datasets , and and additional SNP (rs76895963) was missing just from the COURAGE-PD datasets. These latter two SNPs could not be LD proxied, and they were missing in the COURAGE- PD AAO dataset in the HbA1c arm, too.

COURAGE-PD																				
	PD risk										PD AAO									
	Fasting glucose					HbA1c					Fasting glucose					HbA1c				
Drug	MR method	# SNPs	Beta	Standard error	P	MR method	# SNPs	Beta	Standard error	P	MR method	# SNPs	Beta	Standard error	P	MR method	# SNPs	Beta	Standard error	P
Metformin	Wald ratio	1	-5,155	2,166	0,017						Wald ratio	1	-8,684	15,088	0,565					
Insulin	IVW	2	-1,537	4,263	0,718	Wald ratio	1	5,348	3,994	0,181	IVW	2	-6,018	11,475	0,6	Wald ratio	1	-3,782	27.641	0,891
GLP1	IVW	3	3,024	1,496	0,043	IVW	3	0,538	2,421	0,824	IVW	3	-1,409	10,373	0,892	IVW	3	-5,813	14,337	0,685
GLP1	MR Egger	3	4,47	13,488	0,796	MR Egger	3	28,292	37,704	0,59	MR Egger	3	42,103	93,331	0,73	MR Egger	3	47,409	196,469	0,849
GLP1	Weighted median	3	2,939	1,745	0,092	Weighted median	3	0,288	2,709	0,915	Weighted median	3	-0,573	12,269	0,963	Weighted median	3	-3,607	16,222	0,824
GLP1	Weighted mode	3	2,813	1,944	0,285	Weighted mode	3	0,009	3,238	0,998	Weighted mode	3	1,351	15,097	0,937	Weighted mode	3	-3,138	17,823	0,876
Sulfonylureas	IVW	4	0,014	0,855	0,987	IVW	2	-0,703	1,421	0,621	IVW	4	0,698	2,676	0,794	IVW	2	-0,921	6,295	0,884
Sulfonylureas	MR Egger	4	-1,445	0,561	0,123						MR Egger	4	-0,968	3,884	0,826					
Sulfonylureas	Weighted median	4	-0,118	0,404	0,77						Weighted median	4	0,376	2,661	0,888					
Sulfonylureas	Weighted mode	4	-0,38	0,429	0,441						Weighted mode	4	0,349	2,848	0,91					
TZD	IVW	14	-0,174	0,438	0,691	IVW	18	-0,226	0,618	0,715	IVW	17	0,614	2,27	0,787	IVW	20	-3,226	3,904	0,409
TZD	MR Egger	14	-0,387	0,651	0,563	MR Egger	18	-1,437	1,216	0,255	MR Egger	17	-0,001	3,373	1	MR Egger	20	-1,97	7,898	0,806
TZD	Weighted median	14	-0,296	0,386	0,442	Weighted median	18	-0,62	0,798	0,437	Weighted median	17	0,469	2,663	0,86	Weighted median	20	-0,928	5,326	0,862
TZD	Weighted mode	14	-0,267	0,392	0,508	Weighted mode	18	-0,766	0,757	0,326	Weighted mode	17	0,492	2,663	0,856	Weighted mode	20	-2,739	5,649	0,633

Supplementary table 4. COURAGE-PD stratified MR, unadjusted P. Note: IVW = Inverse variance weighted, TZD = Thiazolidinediones

Footnotes: Of the original 17 IVs (SNPs) for TZD in the FG PD risk arm, three (rs1083864, rs2070737, rs6446479) were filtered out during harmonisation (due to being palindromic with intermediate allele frequencies). Of the original 22 SNPs for TZD in the HbA1c arms, two (rs13232120, rs4894769) were excluded from the PD risk analysis for similar reasons, while two SNPs (rs28678477, rs76895963) were missing from the COURAGE-PD datasets. These latter two SNPs could not be LD proxied.

IPDGC																				
	PD risk										PD AAO									
	Fasting glucose					HbA1c					Fasting glucose					HbA1c				
Drug	MR method	# SNPs	Beta	Standard error	P	MR method	# SNPs	Beta	Standard error	P	MR method	# SNPs	Beta	Standard error	P	MR method	# SNPs	Beta	Standard error	P
Metformin	Wald ratio	1	-1,509	1,596	0,345						Wald ratio	1	-9,07	10,711	0,397					
Insulin	IVW	2	0,561	2,232	0,801	Wald ratio	1	4,667	2,692	0,083	IVW	2	-4,632	7,892	0,557	Wald ratio	1	0,0256	18,244	0,999
GLP1	IVW	3	0,728	1,438	0,613	IVW	3	-1,332	2,828	0,638	IVW	3	6,537	7,147	0,36	IVW	3	7,262	9,563	0,448
GLP1	MR Egger	3	7,023	15,918	0,735	MR Egger	3	25,085	47,826	0,692	MR Egger	3	17,608	74,119	0,852	MR Egger	3	44,272	130,935	0,792
GLP1	Weighted median	3	0,774	1,451	0,594	Weighted median	3	-2,087	2,007	0,298	Weighted median	3	0,956	8,846	0,914	Weighted median	3	7,098	11,297	0,53
GLP1	Weighted mode	3	1,23	2,002	0,602	Weighted mode	3	-2,797	2,332	0,353	Weighted mode	3	0,406	10,576	0,973	Weighted mode	3	7,515	12,761	0,616
Sulfonylureas	IVW	4	0,057	0,584	0,922	IVW	2	0,382	1,053	0,717	IVW	4	2,894	1,842	0,116	IVW	2	4,699	4,311	0,276
Sulfonylureas	MR Egger	4	-0,283	0,981	0,8						MR Egger	4	0,957	2,684	0,756					
Sulfonylureas	Weighted median	4	-0,003	0,29	0,992						Weighted median	4	2,587	1,827	0,157					
Sulfonylureas	Weighted mode	4	-0,044	0,297	0,892						Weighted mode	4	2,338	1,893	0,305					
TZD	IVW	14	0,055	0,295	0,852	IVW	19	0,191	0,502	0,704	IVW	14	2,335	1,655	0,158	IVW	18	5,662	2,775	0,041
TZD	MR Egger	14	-0,009	0,445	0,985	MR Egger	19	-0,314	0,99	0,755	MR Egger	14	3,718	2,418	0,15	MR Egger	18	1,761	5,474	0,752
TZD	Weighted median	14	-0,013	0,275	0,961	Weighted median	19	0,128	0,571	0,822	Weighted median	14	2,403	1,891	0,204	Weighted median	18	4,729	4,039	0,242
TZD	Weighted mode	14	0,006	0,312	0,986	Weighted mode	19	0,095	0,528	0,86	Weighted mode	14	2,592	1,825	0,179	Weighted mode	18	4,102	4,228	0,346

Supplementary table 5. IPDGC stratified MR, unadjusted P. Note: IVW = Inverse variance weighted, TZD = Thiazolidinediones

Footnotes: Of the original 17 IVs in the FG arm, three SNPs were filtered away (rs1083864, rs2070737, rs6446479) during harmonisation (due to being palindromic with intermediate allele frequencies). From the original 22 IVs in the HbA1c arms, two SNPs were removed for similar reasons in the PD risk arm and they were also removed from the IPDGC AAO dataset (rs13232120, rs4894769), while an additional SNP was missing from the IPDGC PD risk dataset (rs28678477), and one more was missing from the IPDGC AAO dataset (rs76895963). These latter two SNPs could not be LD proxied.

	Meta-analysis																			
	PD risk										PD AAO									
	Fasting glucose					Hba1c					Fasting glucose					HbA1c				
Drug	MR method	# SNPs	Beta	Standard error	P	MR method	# SNPs	Beta	Standard error	P	MR method	# SNPs	Beta	Standard error	P	MR method	# SNPs	Beta	Standard error	P
DPP-4	Wald ratio	1	1,317	1,6187	0,416	Wald ratio	1	-0,207	1,692	0,903	Wald ratio	1	-3,788	11,066	0,732	Wald ratio	1	6,816	11,386	0,549
	COURAGE-PD																			
Drug	MR method	# SNPs	Beta	Standard error	P	MR method	# SNPs	Beta	Standard error	P	MR method	# SNPs	Beta	Standard error	P	MR method	# SNPs	Beta	Standard error	P
DPP-4	Wald ratio	1	2,97	2,955	0,315	Wald ratio	1	-1,093	3,265	0,738	Wald ratio	1	-13,022	20,435	0,524	Wald ratio	1	-1,656	22,172	0,94
	IPDGC																			
Drug	MR method	# SNPs	Beta	Standard error	P	MR method	# SNPs	Beta	Standard error	P	MR method	# SNPs	Beta	Standard error	P	MR method	# SNPs	Beta	Standard error	P
DPP-4	Wald ratio	1	0,609	1,935	0,753	Wald ratio	1	0,118	1,978	0,952	Wald ratio	1	0,043	13,163	0,997	Wald ratio	1	9,849	13,269	0,458

Supplementary table 6. Wald ratio MR analysis of DPP-4 inhibitors that failed the positive control

PD risk													
Fasting glucose							HbA1c						
Gene	N SNP	H0	H1	H2	H3	H4	Gene	N SNP	H0	H1	H2	H3	H4
ABCB11	328	0.000	**0.980**	0.000	0.011	0.009	ABCB11	328	0.000	**0.980**	0.000	0.011	0.009
CYP1A2	16	0.097	**0.877**	0.000	0.001	0.026	AGER	21	0.007	**0.933**	0.000	0.001	0.059
IARS1	168	**0.789**	0.203	0.005	0.001	0.002	ALDH8A1	57	0.000	**0.986**	0.000	0.003	0.011
IGF1R	784	0.000	**0.957**	0.000	0.029	0.014	APOC2	40	0.122	**0.868**	0.000	0.001	0.009
INHBE	23	0.203	**0.787**	0.000	0.001	0.010	CDH1	256	0.003	**0.976**	0.000	0.007	0.013
INS	15	0.003	**0.777**	0.000	0.001	0.219	DPAGT1	32	0.232	**0.376**	0.008	0.013	0.371
MAPRE3	57	0.000	**0.858**	0.000	0.005	0.137	IDE	252	**0.858**	0.098	0.037	0.004	0.002
NR1H3	50	0.000	**0.983**	0.000	0.002	0.015	INS	15	0.404	**0.514**	0.001	0.001	0.080
PDIA5	425	0.000	**0.964**	0.000	0.012	0.024	NR1H3	50	0.001	**0.982**	0.000	0.002	0.015
REEP3	207	0.047	**0.925**	0.000	0.008	0.020	PDIA5	425	0.000	**0.964**	0.000	0.012	0.024
SKAP1	597	0.030	**0.939**	0.001	0.016	0.014	PHETA1	17	0.015	**0.968**	0.000	0.001	0.016
SLC2A1	115	**0.702**	0.291	0.003	0.001	0.004	PKLR	20	0.001	**0.978**	0.000	0.003	0.018
TP53INP1	60	0.002	**0.986**	0.000	0.002	0.010	PPIL6	104	**0.580**	0.409	0.003	0.002	0.005
							SLC25A20	40	0.162	**0.802**	0.002	0.009	0.025
							SLC25A26	585	0.135	**0.834**	0.003	0.017	0.011
							SREBF1	38	0.230	**0.681**	0.001	0.003	0.084
							TF	100	**0.701**	0.293	0.002	0.001	0.004
							TMPRSS6	144	0.000	**0.982**	0.000	0.005	0.013

PD AAO													
Fasting glucose							HbA1c						
Gene	N SNP	H0	H1	H2	H3	H4	Gene	N SNP	H0	H1	H2	H3	H4
ABCB11	303	0.000	**0.982**	0.000	0.007	0.011	ABCB11	303	0.000	**0.982**	0.000	0.007	0.011
CYP1A2	16	0.098	**0.894**	0.000	0.000	0.008	AGER	20	0.007	**0.975**	0.000	0.000	0.018
IARS1	155	**0.797**	0.200	0.002	0.000	0.001	ALDH8A1	52	0.000	**0.990**	0.000	0.001	0.009
IGF1R	655	0.000	**0.976**	0.000	0.015	0.009	APOC2	38	0.125	**0.869**	0.000	0.000	0.005
INHBE	17	0.208	**0.785**	0.000	0.000	0.006	CDH1	238	0.004	**0.980**	0.000	0.008	0.009
INS	14	0.003	**0.990**	0.000	0.000	0.007	DPAGT1	32	0.380	**0.616**	0.000	0.000	0.004
MAPRE3	52	0.000	**0.994**	0.000	0.001	0.006	IDE	243	**0.893**	0.100	0.004	0.000	0.001
NR1H3	48	0.000	**0.952**	0.000	0.001	0.047	INS	14	0.443	**0.553**	0.000	0.000	0.004
PDIA5	372	0.000	**0.832**	0.000	0.159	0.009	NR1H3	48	0.001	**0.951**	0.000	0.001	0.047
REEP3	185	0.048	**0.940**	0.000	0.005	0.007	PDIA5	372	0.000	**0.832**	0.000	0.159	0.009
SKAP1	545	0.032	**0.933**	0.000	0.014	0.020	PHETA1	14	0.015	**0.977**	0.000	0.000	0.007
SLC2A1	92	**0.710**	0.287	0.001	0.000	0.002	PKLR	20	0.001	**0.991**	0.000	0.001	0.008
TP53INP1	44.000	0.002	**0.933**	0.000	0.007	0.059	PPIL6	99	0.582	**0.406**	0.002	0.001	0.010
							SLC25A20	40	0.150	**0.740**	0.000	0.001	0.109
							SLC25A26	506	0.143	**0.824**	0.004	0.021	0.008
							SREBF1	37	0.254	**0.739**	0.000	0.001	0.005
							TF	97	**0.704**	0.293	0.001	0.000	0.002
							TMPRSS6	138	0.000	**0.991**	0.000	0.002	0.007

Supplementary table 7. Colocalization. Posterior probabilities for different hypotheses about a shared causal variant between biomarkers FG and HbA1c on the one hand and PD risk (top) and PD age at onset (bottom), for drug clusters metformin and TZD. H0 = no association, H1 = association with biomarker, H2 = association with PD outcome, H3 = association with both biomarker and PD outcome at different locations, H4 = association with both biomarker and PD outcome at the same location. Note: the metformin IV is rs6598541 (gene IGF1R), which is also in the TZD IV set (both of them only appearing in the FG arm). The PD outcomes were calculated using IPDGC data only. Variants were harmonized against the samples from European descent in the 1000 Genome project Phase 3.

Meta-analysis															
PD risk								PD AAO							
Fasting glucose				HbA1c				Fasting glucose				HbA1c			
SNP excluded	Beta	Standard error	P	SNP excluded	Beta	Standard error	P	SNP excluded	Beta	Standard error	P	SNP excluded	Beta	Standard error	P
rs11039154	-0,111	0,293	0,706	rs11039154	-0,006	0,516	0,991	rs11039154	1,02	1,391	0,464	rs11039154	1,95	2,323	0,401
rs12353096	-0,033	0,296	0,912	rs11187019	0,063	0,522	0,904	rs12353096	1,569	1,43	0,273	rs11187019	2,839	2,299	0,217
rs13020526	-0,1	0,269	0,71	rs11708022	0,146	0,518	0,778	rs13020526	1,719	1,467	0,241	rs11708022	1,986	2,312	0,39
rs16954324	-0,023	0,298	0,938	rs12067675	0,138	0,525	0,792	rs16954324	1,623	1,456	0,265	rs12067675	2,7	2,325	0,245
rs28661248	-0,051	0,292	0,862	rs28661248	0,044	0,517	0,932	rs28661248	1,874	1,389	0,177	rs28661248	3,073	2,299	0,181
rs2960192	-0,008	0,296	0,977	rs3130349	0,025	0,521	0,962	rs2960192	1,464	1,39	0,292	rs3130349	2,716	2,312	0,24
rs3729548	-0,025	0,297	0,932	rs3811658	0,087	0,525	0,868	rs3729548	1,582	1,437	0,271	rs3811658	2,877	2,303	0,212
rs3741414	-0,037	0,296	0,902	rs3842756	-0,027	0,493	0,956	rs3741414	1,655	1,46	0,257	rs3842756	2,834	2,308	0,219
rs3811699	0,015	0,288	0,957	rs4820268	0,055	0,577	0,924	rs3811699	1,703	1,463	0,245	rs4820268	4,848	2,529	0,055
rs3842754	-0,089	0,268	0,741	rs61318425	0,129	0,519	0,804	rs3842754	1,708	1,462	0,243	rs61318425	2,345	2,306	0,309
rs557462	0,365	0,536	0,495	rs617948	-0,142	0,386	0,713	rs557462	1,5	2,761	0,587	rs617948	2,924	2,31	0,206
rs6479911	0,027	0,269	0,921	rs7198799	0,126	0,526	0,81	rs6479911	1,795	1,425	0,208	rs7198799	2,553	2,321	0,271
rs6598541	0,045	0,27	0,868	rs7257476	0,086	0,526	0,87	rs6598541	1,927	1,386	0,164	rs7257476	2,592	2,308	0,261
rs896854	-0,049	0,294	0,869	rs7630745	0,089	0,526	0,866	rs896854	2,096	1,354	0,122	rs7630745	2,589	2,309	0,262
None of the 14	-0,025	0,284	0,931	rs7749106	0,2	0,518	0,7	None of the 14	1,671	1,393	0,23	rs7749106	2,425	2,345	0,301
				rs853777	0,3	0,657	0,648					rs853777	2,495	2,919	0,393
				rs874286	0,047	0,523	0,929					rs874286	3,089	2,312	0,181
				rs9894257	0,223	0,481	0,644					rs9894257	2,804	2,312	0,225
				None of the 18	0,083	0,504	0,869					None of the 18	2,745	2,282	0,229
COURAGE-PD															
PD risk								PD AAO							
Fasting glucose				HbA1c				Fasting glucose				HbA1c			
SNP excluded	Beta	Standard error	P	SNP excluded	Beta	Standard error	P	SNP excluded	Beta	Standard error	P	SNP excluded	Beta	Standard error	P
rs11039154	-0,29	0,455	0,524	rs11039154	-0,35	0,625	0,575	rs1083864	0,434	2,324	0,852	rs11039154	-3,741	3,967	0,346
rs12353096	-0,171	0,459	0,71	rs11187019	-0,275	0,635	0,665	rs11039154	0,308	2,345	0,895	rs11187019	-3,122	3,933	0,427
rs13020526	-0,192	0,461	0,678	rs11708022	-0,229	0,646	0,722	rs12353096	0,316	2,283	0,89	rs11708022	-3,588	3,953	0,364
rs16954324	-0,163	0,459	0,722	rs12067675	-0,252	0,647	0,697	rs13020526	0,763	2,298	0,74	rs12067675	-3,021	3,968	0,446
rs28661248	-0,233	0,446	0,601	rs28661248	-0,319	0,622	0,607	rs16954324	0,897	2,286	0,695	rs13232120	-2,923	3,951	0,459
rs2960192	-0,239	0,437	0,585	rs3130349	-0,168	0,643	0,794	rs2070737	0,449	2,29	0,844	rs28661248	-2,81	3,94	0,476
rs3729548	-0,148	0,455	0,744	rs3811658	-0,181	0,638	0,776	rs28661248	0,911	2,292	0,691	rs3130349	-2,414	3,964	0,543
rs3741414	-0,25	0,418	0,551	rs3842756	-0,345	0,61	0,572	rs2960192	0,34	2,287	0,882	rs3811658	-3,243	3,939	0,41
rs3811699	-0,126	0,449	0,779	rs4820268	-0,355	0,703	0,613	rs3729548	0,446	2,283	0,845	rs3842756	-3,215	3,944	0,415
rs3842754	-0,241	0,437	0,581	rs61318425	-0,183	0,64	0,775	rs3741414	0,75	2,283	0,742	rs4820268	-2,321	4,293	0,589
rs557462	0,391	0,842	0,642	rs617948	-0,505	0,585	0,388	rs3811699	0,365	2,289	0,873	rs4894769	-3,638	3,958	0,358
rs6479911	-0,111	0,43	0,796	rs7198799	-0,254	0,647	0,695	rs3842754	0,664	2,289	0,772	rs61318425	-3,27	3,942	0,407
rs6598541	-0,047	0,399	0,906	rs7257476	-0,175	0,641	0,785	rs557462	1,203	3,919	0,759	rs617948	-3,151	3,949	0,425
rs896854	-0,223	0,45	0,621	rs7630745	-0,173	0,639	0,787	rs6446479	0,627	2,288	0,784	rs7198799	-3,241	3,967	0,414
None of the 14	-0,174	0,438	0,691	rs7749106	-0,038	0,628	0,951	rs6479911	0,722	2,283	0,752	rs7257476	-3,591	3,951	0,363
				rs853777	0,272	0,784	0,729	rs6598541	0,829	2,296	0,718	rs7630745	-3,227	3,948	0,414
				rs874286	-0,226	0,646	0,727	rs896854	0,785	2,29	0,732	rs7749106	-3,761	4,018	0,349
				rs9894257	-0,14	0,63	0,825	None of the 17	0,614	2,27	0,787	rs853777	-4,639	4,898	0,344
				None of the 18	-0,226	0,618	0,715					rs874286	-2,955	3,955	0,455
												rs9894257	-3,027	3,949	0,443
												None of the 20	-3,226	3,904	0,409
IPDGC															

PD risk								PD AAO							
Fasting glucose				HbA1c				Fasting glucose				HbA1c			
SNP excluded	Beta	Standard error	P	SNP excluded	Beta	Standard error	P	SNP excluded	Beta	Standard error	P	SNP excluded	Beta	Standard error	P
rs11039154	-0,015	0,312	0,963	rs11039154	0,121	0,518	0,816	rs11039154	1,549	1,69	0,359	rs11039154	4,755	2,826	0,092
rs12353096	0,041	0,306	0,895	rs11187019	0,184	0,52	0,724	rs12353096	2,34	1,731	0,177	rs11187019	5,747	2,796	0,04
rs13020526	-0,051	0,253	0,84	rs11708022	0,282	0,505	0,577	rs13020526	2,335	1,746	0,181	rs11708022	4,725	2,813	0,093
rs16954324	0,051	0,309	0,869	rs12067675	0,282	0,513	0,582	rs16954324	2,121	1,65	0,199	rs12067675	5,506	2,83	0,052
rs28661248	0,045	0,308	0,883	rs28661248	0,177	0,518	0,732	rs28661248	2,481	1,7	0,144	rs28661248	5,921	2,794	0,034
rs2960192	0,114	0,285	0,689	rs3130349	0,075	0,494	0,879	rs2960192	2,175	1,703	0,201	rs3130349	5,186	2,808	0,065
rs3729548	0,04	0,306	0,897	rs3811658	0,173	0,52	0,739	rs3729548	2,292	1,727	0,185	rs3811658	5,864	2,801	0,036
rs3741414	0,077	0,306	0,801	rs3842756	0,087	0,495	0,86	rs3741414	2,247	1,723	0,192	rs3842756	5,795	2,808	0,039
rs3811699	0,091	0,303	0,764	rs4820268	0,202	0,571	0,723	rs3811699	2,515	1,702	0,139	rs4820268	8,347	3,08	0,007
rs3842754	-0,007	0,285	0,98	rs61318425	0,235	0,516	0,648	rs3842754	2,369	1,738	0,173	rs61318425	5,095	2,805	0,069
rs557462	0,352	0,561	0,53	rs617948	-0,001	0,43	0,998	rs557462	2,154	3,288	0,512	rs617948	5,893	2,811	0,036
rs6479911	0,1	0,289	0,73	rs7198799	0,265	0,516	0,608	rs6479911	2,466	1,703	0,148	rs7198799	5,391	2,824	0,056
rs6598541	0,094	0,303	0,756	rs7257476	0,168	0,52	0,747	rs6598541	2,605	1,666	0,118	rs7257476	5,613	2,81	0,046
rs896854	0,044	0,309	0,888	rs7630745	0,172	0,522	0,742	rs896854	2,876	1,643	0,08	rs7630745	5,435	2,809	0,053
None of the 14	0,055	0,295	0,852	rs76895963	0,24	0,522	0,645	None of the 14	2,335	1,655	0,158	rs7749106	5,435	2,848	0,056
				rs7749106	0,264	0,522	0,613					rs853777	6,103	3,559	0,086
				rs853777	0,231	0,652	0,723					rs874286	6,03	2,811	0,032
				rs874286	0,136	0,515	0,792					rs9894257	5,658	2,818	0,045
				rs9894257	0,35	0,466	0,453					None of the 18	5,662	2,775	0,041

Supplementary table 8. Leave-one-out MR analysis of TZD using IVW

Drug	Class	Number of genes	PUBCHEM	DGIDB
2,4-THIAZOLIDINEDIONE	Thiazolidinediones	15	x	
ALOGLIPTIN	DPP4	12	x	
ALOGLIPTIN	DPP4	1		x
BEXAGLIFLOZIN	SGLT2	8	x	
BEXAGLIFLOZIN	SGLT2	1		x
CANAGLIFLOZIN	SGLT2	18	x	
CANAGLIFLOZIN	SGLT2	2		x
CHLORPROPAMIDE	Sulfonylureas	34	x	
CHLORPROPAMIDE	Sulfonylureas	11		x
DAPAGLIFLOZIN PROPANEDIOL	SGLT2	2	x	
DAPAGLIFLOZIN PROPANEDIOL	SGLT2	2		x
DULAGLUTIDE	GLP1	1		x
EMPAGLIFLOZIN	SGLT2	19	x	
EMPAGLIFLOZIN	SGLT2	2		x
ERTUGLIFLOZIN	SGLT2	18	x	
ERTUGLIFLOZIN	SGLT2	2		x
EXENATIDE	GLP1	25	x	
EXENATIDE	GLP1	1		x
GLIMEPIRIDE	Sulfonylureas	43	x	
GLIMEPIRIDE	Sulfonylureas	8		x
GLIPIZIDE	Sulfonylureas	28	x	
GLIPIZIDE	Sulfonylureas	9		x
GLYBURIDE	Sulfonylureas	106	x	
GLYBURIDE	Sulfonylureas	26		x
INSULIN ASPART PROTAMINE, HUMAN	Insulin	1		x
INSULIN DEGLUDEC	Insulin	1	x	
INSULIN DEGLUDEC	Insulin	2		x
INSULIN DETEMIR	Insulin	3	x	
INSULIN DETEMIR	Insulin	1		x
INSULIN GLARGINE	Insulin	4	x	
INSULIN GLARGINE-YFGN	Insulin	1		x
INSULIN GLULISINE, HUMAN	Insulin	1		x
INSULIN LISPRO-AABC	Insulin	1		x
LINAGLIPTIN	DPP4	14	x	
LINAGLIPTIN	DPP4	2		x
LIRAGLUTIDE	GLP1	19	x	
LIRAGLUTIDE	GLP1	2		x
LIXISENATIDE	GLP1	1	x	
LIXISENATIDE	GLP1	1		x
METFORMIN	Metformin	67	x	
METFORMIN	Metformin	36		x
PIOGLITAZONE	Thiazolidinediones	375	x	

PIOGLITAZONE HYDROCHLORIDE	Thiazolidinediones	16		x
ROSIGLITAZONE	Thiazolidinediones	1229	x	
ROSIGLITAZONE	Thiazolidinediones	23		x
SAXAGLIPTIN	DPP4	15	x	
SAXAGLIPTIN ANHYDROUS	DPP4	1		x
SEMAGLUTIDE	GLP1	3	x	
SEMAGLUTIDE	GLP1	1		x
SITAGLIPTIN	DPP4	25	x	
SITAGLIPTIN	DPP4	4		x
TOLAZAMIDE	Sulfonylureas	18	x	
TOLAZAMIDE	Sulfonylureas	7		x
TOLBUTAMIDE	Sulfonylureas	76	x	
TOLBUTAMIDE	Sulfonylureas	6		x

Supplementary table 9. Displaying the antidiabetic drugs retained from the resources and the number of genes associated, and the drug classes of the drugs.

Traits	Type of study	Data source	Publication DOI	Participants	Web source	Build	Downloaded	Comments
Fasting glucose	GWAS summary statistics	MAGIC	10.1038/s41588-021-00852-9	200 622 participants of European heritage	https://magicinvestigators.org/downloads/	GRCh37	2023-07-21	
HbA1c	GWAS summary statistics	MAGIC	10.1038/s41588-021-00852-9	146 806 participants of European heritage	https://magicinvestigators.org/downloads/	GRCh37	2023-07-21	
0	Multi-tissue eQTL	GTEx	10.1038/ng.2653<br><br>10.1016/j.xhgg.2021.100083	48 non-diseased tissue sites across at least 70 individuals	https://www.gtexportal.org/home/downloads/adult-gtex/qtl	GRCh37	2024-06-19	V7
Type 2 diabetes (wide definition)	GWAS summary statistics	FinngGen	10.1038/s41586-022-05473-8	38 657 participants of Finnish (European) heritage	https://elomake.helsinki.fi/lomakkeet/124935/lomake.html	GRCH38	2025-03-26	
Childhood asthma	GWAS summary statistics	UK Biobank	10.1186/s13059-020-02248-0	1 993 cases and 359 201 controls of European heritage	https://gwas.mrcieu.ac.uk/datasets/ukb-d-ASTHMA_CHILD/	GRCh37	2025-02-06	
PD risk	GWAS summary statistics	COURAGE-PD	unpublished yet, but other study using this resource: 10.1212/WNL.0000000000209620 preprint: 10.1101/2024.08.21.24311915	8409 cases and 8 034 controls of predominantly European ancestry	Not publicly available	GRCh37	2024-09-16	Excluding IPDGC overlap
PD risk	GWAS summary statistics	IPDGC	10.1016/S1474-4422(19)30320-5	33 674 cases and 449 056 controls	https://drive.google.com/drive/folders/10bGj6HfAXgl-JslpI9ZJIL_JIgZyktxn	GRCh37	2024-08-27	Excluding 23andMe
PD AAO	GWAS summary statistics	COURAGE-PD	10.1212/WNL.0000000000200699	8 535 PD cases of predominantly European ancestry	Not publicly available	GRCh37	2024-09-16	Excluding IPDGC overlap
PD AAO	GWAS summary statistics	IPDGC	10.1002/mds.27659	28 568 PD cases	Not available currently 2025-04-25	GRCh37	2024-06-24	Excluding 23andMe

Supplementary table 10. GWAS and eQTL resources used in the study

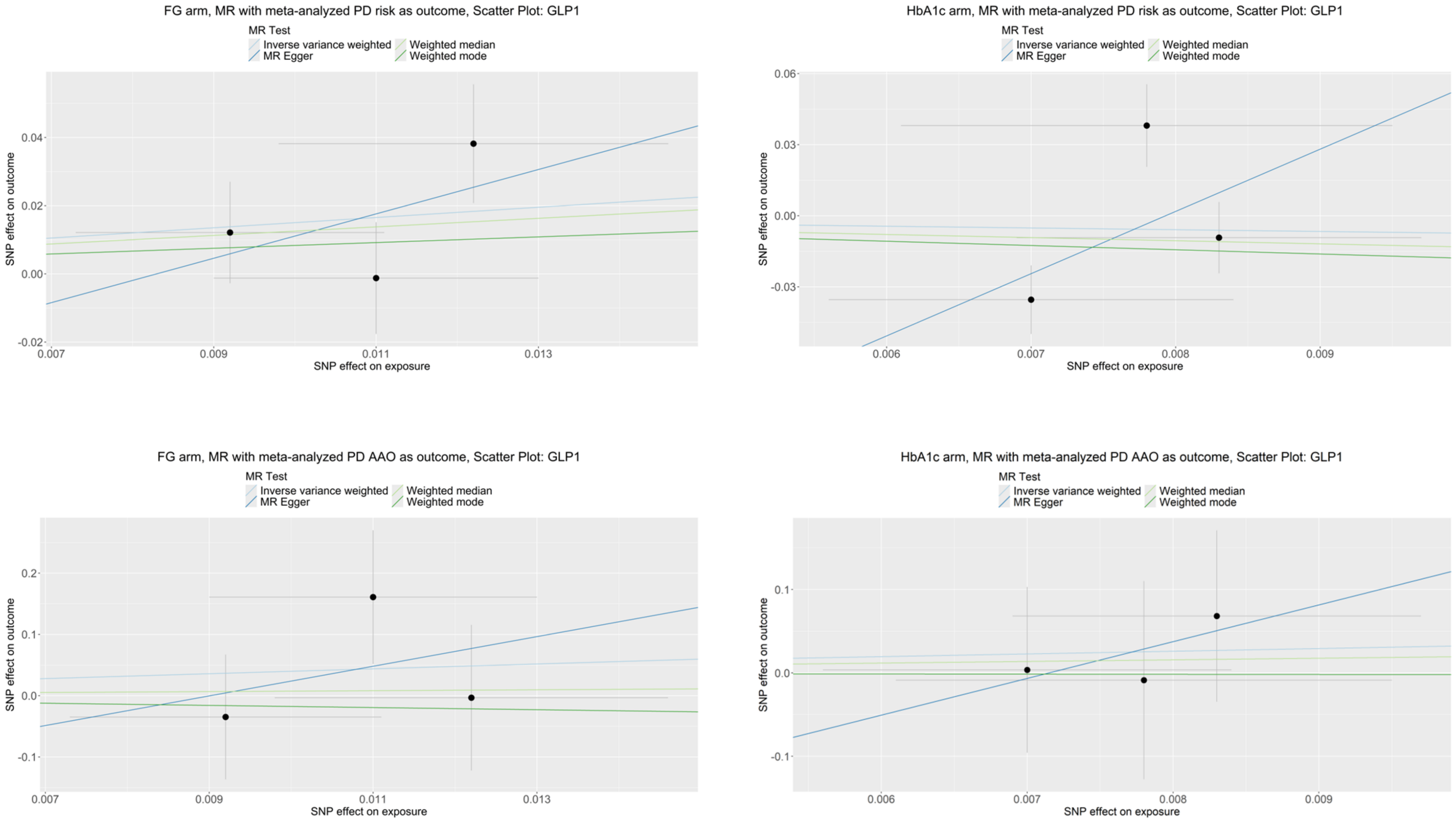

Supplementary figure 1. GLP-1 receptor agonist MR models scatterplots, posthoc sensitivity analysis

Checklist for reviewing Mendelian randomization investigations

1. What is the primary hypothesis of interest? What is the motivation for using Mendelian randomization? What is the scope of the investigation? What and how many primary analyses?

2. Data sources
   What type of Mendelian randomization investigation is this? One-sample or two-sample? Sample overlap? Summarized data or individual-level data? Drawn from same population? Relevance to applied research?

3. Selection of genetic variants – how were the genetic variants chosen? Single or multiple gene regions?
   a. Biological rationale?
   b. GWAS analysis? If so, what dataset? What was the p-value threshold? Clumping?
   c. Were genetic variants excluded from the analysis? Associations with pleiotropic pathways?
   d. How else was the validity of genetic variants as instrumental variables assessed?

4. Variant harmonization (for two-sample analyses)
   Was it checked that genetic variants were appropriately orientated across the datasets?

5. Primary analysis
   What was the primary analysis? What was the statistical method? How implemented? Multiple testing?

6 and 7. Supplementary and sensitivity analyses
   What analyses were performed to support and assess the validity of the primary analysis?
   For example: stricter criteria for variant selection, assess heterogeneity, robust methods, subgroup analysis, positive/negative controls, 'leave-one-out' analyses, colocalization (single gene region)

8. Extensions and additional analyses
   What additional analyses were performed to better understand the nature of the causal effect?

9. Data presentation
   How are the data and results presented to allow readers to assess the analysis and assumptions?
   For example: scatter plot, forest/funnel/radial plot, R^2/F statistics, comparison of methods, power

10. Interpretation
    How have results been interpreted, particularly any numerical estimates?

Burgess S, Davey Smith G, Davies NM et al. Guidelines for performing Mendelian randomization investigations: update for summer 2023 [version 3]. Wellcome Open Res 2023, 4:186 (doi: 10.12688/wellcomeopenres.15555.3)

Wellcome Open Research

Supplementary figure 2. MR Guidelines from https://wellcomeopenresearch.org/articles/4-186/v3
